# Supercritical Carbon Dioxide in Presence of Water for the Valorization of Spent Coffee Grounds: Optimization by Response Surface Methodology and Investigation of Caffeine Extraction Mechanism

**DOI:** 10.3390/foods11244089

**Published:** 2022-12-17

**Authors:** Alexandre Vandeponseele, Micheline Draye, Christine Piot, Damien Bernard, Philippe Fanget, Gregory Chatel

**Affiliations:** EDYTEM, University Savoie Mont Blanc, CNRS, F-73000 Chambéry, France

**Keywords:** supercritical CO_2_, extraction, spent coffee grounds, design of experiments, lipids, polar molecules fraction, caffeine, mechanism

## Abstract

Spent coffee grounds are a promising bioresource that naturally contain around 50 wt% moisture which requires, for a valorization, a drying step of high energy and economic costs. However, the natural water in spent coffee grounds could bring new benefits as a co-solvent during the supercritical CO_2_ extraction (SC-CO_2_). This work reports the influence and optimization of pressure (115.9–284.1 bars), temperature (33.2–66.8 °C), and moisture content (6.4–73.6 wt%) on simultaneous extraction of lipids and polar molecules contained in spent coffee grounds by supercritical CO_2_ (SC-CO_2_) using Central Composite Rotatable Design and Response Surface Methodology. The results show that for lipids extraction, pressure is the most influent parameter, although the influence of moisture content is statistically negligible. This suggests that water does not act as barrier to CO_2_ diffusion in the studied area. However, moisture content is the most influent parameter for polar molecules extraction, composed of 99 wt% of caffeine. Mechanism investigations highlight that H_2_O mainly act by (i) breaking caffeine interactions with chlorogenic acids present in spent coffee grounds matrix and (ii) transferring selectively caffeine without chlorogenic acid by liquid/liquid extraction with SC-CO_2_. Thus, the experiment for the optimization of lipids and polar molecules extraction is performed at a pressure of 265 bars, a temperature of 55 °C, and a moisture content of 55 wt%.

## 1. Introduction

Overconsumption of natural resources is one of the major issues of the 21st century, symbolized by the continuous increase of Ecological Debt Day or Earth Overshoot Day, which was on the 28 July 2022 [1]. One solution might come from the circular economy concept, which suggests turning organic waste into additional renewable resources [2]. Several biomasses of “waste” type have been studied in recent years such as orange or potato peel [3,4], apple, grape, or olive pomace [5,6,7], grape winery waste [8], spent brewer’s grains [9], or spent coffee grounds [10] for the production of high value polyphenols and oil. Among them, spent coffee grounds (SCG) is one of the most promising bioresource, since each ton of coffee beans generates 650 kg of SCG [11]. Around 6 million tons of SCG are produced worldwide each year [12]. Interestingly, SCG is composed of high value compounds, and in particular 45 to 50% carbohydrates, 10 to 15% lipids, 7 to 13% proteins, 0.5 to 3% chlorogenic acids, and 0 to 0.5% caffeine (*w*/*w*), depending on the variety of coffee (*Coffea arabica, Coffea canephora)*, its geographical brewing stage, or storage conditions [13]. The processes for the recovery of these molecules must be economically and environmentally acceptable, in accordance with the twelve principles of green chemistry and green engineering [14,15]. Green chemistry applied to the field of biomass valorization can rely on innovative processes based on the use of ultrasound (US) [16,17,18,19], microwaves (MW) [20,21], pulse electric fields (PEF) [22], high voltage electrical discharges (HVED) [23,24], subcritical H_2_O (SCW) [10,25], and supercritical CO_2_ (SC-CO_2_) [10,26].

Under conditions of temperature and pressure above the critical point (i.e., 31.1 °C and 73.8 bars), the CO_2_ enters in a supercritical state. In the supercritical state, CO_2_ possesses hybrid properties between gas and liquid. It has a viscosity between 0.02 and 0.12 mPa·s, close to that of a gas, a density between 700 and 1100 kg·m^−3^, close to that of liquid, with a diffusivity power very high compared to that of a liquid fluid at 40–210 °C and 90–500 bars [27,28]. The supercritical CO_2_ processes offer several advantages. Indeed, the use of supercritical CO_2_ does not generate any effluent and CO_2_ is a recyclable, non-toxic, non-flammable, and cheap fluid.

In a review, Vandeponseele et al. investigated the potential to use supercritical CO_2_ for the production of high value molecules such as lipids, caffeine, and polyphenols from spent coffee grounds [10]. Since supercritical CO_2_ is a nonpolar solvent, it is not suitable for the recovery of polar molecules like polyphenols without the addition of a polar modifier or co-solvent. In that context, Araujo et al. showed that SC-CO_2_ with EtOH co-solvent is more efficient than pure SC-CO_2_ for the extraction of phenolic compounds [29].

In all cases, supercritical CO_2_ extractions are usually carried out with dry spent coffee grounds, which are dried before the storage step to prevent fungi development that can lead to the degradation of valuable molecules [30,31]. In addition, wet basis moisture content above 50 wt% can act negatively as a barrier to the diffusion of supercritical CO_2_ into the matrix during biomass extraction [32]. However, the drying step consumes energy, inducing an additional cost to the entire biomass recovery process [33]. Short-time storage, less than two weeks, of spent coffee grounds with high moisture content does not significantly degrade high-added value molecules such as caffeine [31]. Mouahid et al. pointed out that important wet basis water content in algae up to 23% does not lead to barrier diffusion phenomenon during SC-CO_2_ extraction [34]. In addition, they demonstrated the benefits of moisture as a co-solvent that is naturally present in the matrix for the extraction of carotenoids from this algae [34].

Due to the brewing of coffee, spent coffee grounds naturally contain around 50 wt% wet basis moisture. Thus, this work evaluates, for the first time, the negative and positive effects of moisture content of spent coffee grounds during supercritical CO_2_ extraction. To do this, the main objectives of this study are (i) to demonstrate the influence of pressure, temperature, and moisture content of the SCG on the extraction of lipids and polar molecules, (ii) to optimize the process of co-extraction of lipids and phenolic compounds, and (iii) to explain the mechanisms involved in the supercritical CO_2_ extraction of high-added value molecules contained in spent coffee grounds with high moisture content.

## 2. Materials and Methods

### 2.1. Chemicals and Reagents

Pure standard of caffeine, myristic acid (99%), palmitic acid (99%), acetyl chloride (98%), trolox (97%), and ethanol (96 vol%, not denaturated) were obtained from ACROS ORGANICS. Acetonitrile, ethyl acetate, and potassium hydroxide were supplied by Fisher Chemical (Waltham, MA, USA). Pure standard of 3-caffeoylquinic acid (CAS 327-97-9), oleic acid (99%), stearic acid (98%), Vitamin E acetate (97%), DPPH (95%), and cyclohexane were purchased from Alfa Aesar (Haverhill, MA, USA). Caprylic acid (99%) and arachidic acid (99%) were obtained from Sigma Aldrich (St. Louis, MO, USA). Capric acid (99%) and lauric acid (99%) were obtained from Interchim (Montlucon, France). Methanol (≥99.9%) was obtained from Honeywell (Nawabash, IN, USA). Hydrochloric acid (37%) was obtained from Roth. Linoleic acid (99%), and n-hexane (>99.9%) were obtained from Fluka. All solvents and reagents were of analytical grade and used as received.

### 2.2. Biomass Preparation

Spent coffee grounds (SCG) were collected from a local coffee shop (Le-Bourget-du-Lac, France). Their wet basis moisture content after collection was measured up to 53.8 ± 0.6 wt%. SCG was pre-dried in the oven at a low temperature of 50 °C, to prevent thermal degradation, during 48 h. Then, the dried SCG was stored in a freezer at −8 °C. A single batch of SCG was used throughout this study. The drying was performed in this study in order to (i) to define the moisture content in spent coffee grounds and (ii) to prepare samples of spent coffee grounds with controlled moisture content (6.4–73.6 wt%). Before supercritical extractions, the SCG was moistened to the desired moisture content (cf Section 2.3.3) by adding liquid water that was mixed thanks to a spatula until homogenization of the raw material.

Before SC-CO_2_-based extraction with supercritical CO_2_, the frozen SCG was lyophilized for 72 h in a lyophilizer, Buchi Lyovapor L-200, at a pressure of 0.5 mbar and a temperature of −55 °C. The freeze-dried SCG were considered as dry spent coffee grounds and stored in a desiccator between each extraction.

### 2.3. Supercritical CO_2_ Experiments

#### 2.3.1. Supercritical CO_2_ Apparatus

The scheme and description of supercritical CO_2_ apparatus used in this study are presented in Supplementary Information (Figure S1).

#### 2.3.2. General Procedure

Each 60 min experiment was performed with a constant flow rate of CO_2_ of 50 g_CO2_·min^−1^ as follows: 25 g of spent coffee grounds were exposed to different pressure, temperature, and moisture content according to the Design of Experiments, as described in Section 2.3.3. At the end of the extraction, the apparatus was washed two times with 25 mL of ethanol (96 vol%) at 150 bars and 40 °C during 30 min. The raw extract and washing ethanol were gathered before separation into lipids and polar molecules fractions, as described in Section 2.4.

#### 2.3.3. Design of Experiments (DoE)

Design of Experiments (DoE) was used with a Central Composite Rotatable Design (CCRD), a factorial design that efficiently fits with response surface according to the literature [35,36,37,38]. Response Surface Methodology (RSM) is a mathematical and statistical tool of optimization that was used in this study to optimize experimental conditions of supercritical CO_2_ extraction of (i) lipids, (ii) polar molecules, and (iii) of co-extraction of lipids and polar molecules from SCG [35,39]. In this work, the RSM is based on the design of three significant parameters with five levels: pressure (115.9–284.1 bars), temperature (33.2–66.8 °C), and dry basis moisture content (6.4–73.6 wt%), which corresponds to a range of wet basis moisture content of 6.0–42.4 wt%. The range and levels of those three independent variables are presented in Table 1.

The response variables are referred to as Recovery Yield of Lipids (wt%) and Recovery Yield of Polar molecules (wt%). The design was recorded a total of 45 experiments, subdivided in 24 cubic points, 18 axial points, and 3 center points. Those experiments were randomized in order to minimize the effects of a possible unexpected variability. The data from CCRD were analyzed by a multiple regression method to fit a second-order polynomial regression model containing the coefficients for linear, quadratic, and two factor interaction effects. The model equation of response (Y) with three independent variables (*i, j, k*) is described by the following equation (Equation (1)):

Equation (1). Second order polynomial equation of response (Y) with three independent variables (*i, j, k*)

(1) $$Y=\beta_{0}+\overset{3}{\underset{i=1}{\sum }}\beta_{i}+\overset{3}{\underset{i=1}{\sum }}\beta_{ii}x_{i}^{2}+\sum_{i=1}^{2}\overset{3}{\underset{j=i+1}{\sum }}\beta_{ij}x_{i}\;x_{j}$$

where *Y* is the response variable, $\beta_{0}$ is the constant coefficient, $\beta_{i}$ is the linear coefficient, $\beta_{ii}$ is the quadratic coefficient, and $\beta_{ij}$ is the two factors interaction coefficient. The accuracy of the model was determined with the analysis of variance (ANOVA), including the evaluation of the lack of fit, coefficient of determination (R2), and the Fisher test value (F-value). All statistical calculations were made for a confidence level superior at 95% (i.e., with *p* < 0.05). RSM and ANOVA results were generated by the Minitab 17 software (Pennsylvania, PA, USA). Student’s tests for the kinetics of extractions were carried out with the software R (4.0.3 version).

### 2.4. Separation of Raw Extract by Liquid/Liquid Extraction into Lipids and Polar Molecules Fractions

At the end of the process, the raw extract obtained after SC-CO_2_ extraction and ethanol washing was composed of a mixture of immiscible lipids and hydroalcoholic solution enriched in phenolic compounds. Additionally, the separation of the extract was carried out by liquid/liquid extraction of the lipid fraction with 2 unt × 20 mL of n-hexane. After a third wash with 20 mL of n-hexane, 20 mL of water was added to the crude extract to increase the polarity of the hydroalcoholic phase and remove the last traces of lipids.

The n-hexane solution with lipids fraction was then dried with MgSO_4_ before filtration and evaporation at 40 °C and under 250 mbar (Rotavapor R-314, Buchi). Traces of organic solvent in the oil extract were removed by drying with nitrogen flow until the mass remains stable. Then, the oil was weighed, transferred into a sealed flask under N_2_ atmosphere, and stored in the freezer at −8 °C before complete characterization.

The fraction of hydroalcoholic solution containing polar molecules was partially evaporated at 40 ° C under 100 mbar in order to remove the ethanol. 100 mL of H_2_O were then added, and the resulting solution was frozen in the freezer. The frozen extract was lyophilized under 0.5 mbar and at −55 °C for 72 h, and then weighed and stored in the refrigerator at 3 °C before complete characterization.

### 2.5. Analyses of Lipids Fraction

#### 2.5.1. Absolute and Recovery Yield of Lipids

The Absolute Yield of Lipids (Equation (2)) represents the mass of nonpolar fraction extracted after liquid/liquid extraction of the raw extract obtained by supercritical CO_2_ extraction compared to the reference with n-hexane Soxhlet [40]. The Recovery Yield of Lipids (RYL, Equation (3)) represents the mass of oil in nonpolar fraction after liquid/liquid extraction of the raw extract obtained by supercritical CO_2_ extraction in 100 g of dry SCG per mass of oil extracted by n-hexane Soxhlet in 100 g of dry SCG, a reference method to determine oil content [41,42]. The Soxhlet experiment was performed with a cartridge containing 10 g of SCG and 100 mL of n-hexane under 4 h reflux to exhaust the material. The resulting n-hexane fraction was treated in the same way as in Section 2.4 to obtain a dry oil. The term of recovery yield of lipids determined has the advantage of being comparable with those of various studies due to the comparison with the same n-hexane Soxhlet extraction reference, despite the different proportions of lipids present in different matrices.

Equation (2). Absolute Yield of Lipids.

(2) $$Absolute\;Yield\;of\;Lipids\;\left(\%\right)=\frac{mass\;of\;oil\;\;extracted\;\left(g\right)}{mass\;of\;dry\;SCG\;before\;extraction\;\left(g\right)\;}\times 100$$

Equation (3). Recovery Yield of Lipids

(3) $$Recovery\;Yield\;of\;Lipids\;\left(RYL\right)\;\left(\%\right)=\frac{mass\;of\;oil\;extracted\;by\;SC-CO_{2}\;\left(g\cdot 100\;g^{-1}\;\right)}{mass\;of\;oil\;extracted\;by\;hexane\;Soxhlet\;\left(g\cdot 100\;g^{-1}\right)}\times 100$$

#### 2.5.2. Fatty Acids Profile and Level

Fatty acids were transesterified into Fatty Acid Methyl Esters (FAME) by mixing 5 mg of C8:0–C20:0 standards or SCG oil with 1 mL of methanol/acetyl chloride (95/5 *v*/*v*) at 50 °C under stirring at 500 rpm for 8 h [43]. After the transesterification, liquid/liquid extraction was carried out by adding to the mixture 1 mL of H_2_O and 10 mL of n-hexane to recover FAME before their analysis.

GC-MS analytical protocols were modified and adapted from the Campo et al. method [44]. The full protocol and equations of Fatty Acids Profile (FAP) expressed in g_Cx:y_·100 g^−1^ allfattyacids and Fatty Acids Level (FAL) in g_gallfattyacids_·100 g^−1^ oil are detailed in Supplementary Information (See Supplementary Information).

#### 2.5.3. Acid, Saponification, Ester, and Iodine Values (AV, SV, EV, IV)

Acid Value (AV) was measured using the reference method from the ISO 660 protocol [45,46]. Saponification Value (SV) was measured with reference method from the A.O.A.C 920.160 protocol [46,47]. Iodine value (IV) was measured with the reference method according to A.O.A.C 920.159 protocol, modified by using cyclohexane instead of carbon tetrachloride [46,48,49].

The equations of Acid Value (AV), Saponification Value (SV), Ester Value (EV), wt% Free Fatty Acids (%FFA), and Iodine Value (IV) are reported in Supplementary Information (See Supplementary Information).

#### 2.5.4. Viscosity, Density, and Refractive Index

The densities at 20 and 40 °C were determined with a Mettler Toledo DM 45 DeltaRange densitymeter equipped with a Mettler Toledo DryPal Drying Pump. A glass capillary of 1 mL was filled with oil using a syringe. The capillary was washed with ethyl acetate and then with acetone before drying.

The dynamic and kinetic viscosities at 40 °C were determined with an Anton Paar Lovis 2000 M rolling ball microviscosimeter. The apparatus was set with a glass capillary (internal diameter 1.8 mm, length 140 mm, serial number 20644208) and steel ball (internal diameter 1.5 mm, Mat N° 73109, steel 1.4125) with a density of 769 kg·m^−3^. Measurements were carried out with a slope of 45 ° at 40 °C after calibration with Vaseline oil (Chimie Plus, 33009), for which the density is 836 kg·m^−3^ and the kinematic viscosity is 20 mm^2^·s^−1^ at 40 °C.

The refractive index at 20 °C was determined with an Anton Paar Abbemat 550 refractometer. One millimeter of sample was deposited on the glass surface.

#### 2.5.5. AntiOxidant Capacity–DPPH (AOC-DPPH)

The AOC-DPPH was determined in Trolox Equivalent Antioxidant Capacity (TEAC) according to modified Espin et al. method [50,51]. Briefly, 25 µL (20 mg) lipids were mixed with 4500 µL of DPPH solution at concentration 93 µM (36.7 mg·L^−1^) and 475 µL ethyl acetate. The mixture was kept in the dark for one hour before reading an absorbance at λ = 515 nm with an UV-visible spectrophotometer (Varian Cary 50, France).

### 2.6. Analyses of Polar Molecules Fraction

#### 2.6.1. Absolute and Recovery Yield of Polar Molecules

The Absolute Yield of Polar molecules (Equation (4)) represents the mass of polar molecules fraction after separation by liquid/liquid extraction of the raw extract obtained by supercritical CO_2_ extraction per mass of dry SCG [52,53]. Unfortunately, the amount of polar molecules, including polyphenols and caffeine, strongly depends of the raw material composition [54].

The Recovery Yield of Polar molecules (RYP, Equation (5)) represents the mass of polar molecules fraction after separation by liquid/liquid extraction of the raw extract obtained by supercritical CO_2_ compared to the mass of extract with hydroalcoholic solution EtOH/H_2_O (40/60, *v*/*v*). Contrary to lipids case, there is no reference method in the literature to produce polar molecules extract from SCG; thus, the reference method is based on a previous optimization [31]. The reference extraction for polar molecules was conducted on 2 g of defatted SCG with 50 mL of hydroalcoholic solution EtOH/H_2_O (40/60%, *v*/*v*) at 60 °C for 15 min. This reference extraction for polar molecules, including caffeine and polyphenols, was repeated three times with the same defatted SCG to exhaust the raw material in order to define the maximum concentration of polar molecules in SCG. The hydroalcoholic solution was treated according to the protocol described in Section 2.4 to obtain a dry extract.

Equation (4). Absolute Yield of Polar molecules

(4) $$Absolute\;Yield\;of\;Polar\;molecules\;\left(\%\right)=\frac{mass\;of\;polyphenols\;\;extracted\;\left(g\right)}{mass\;of\;dry\;SCG\;before\;extraction\;\left(g\right)}\times 100$$

Equation (5). Recovery Yield of Polar molecules

(5) $$Recovery\;Yield\;of\;Polar\;molecules\;\left(RYP\right)\;\left(\%\right)=\frac{masss\;of\;polyphenols\;extracted\;by\;SC-CO_{2}\;\left(g\cdot 100\;g^{-1}\;\right)}{mass\;of\;polyphenols\;extracted\;by\;hydroalcoholic\;sol\;\left(g\cdot 100\;g^{-1}\right)}\times 100$$

#### 2.6.2. Caffeine, 3-CQA, and Total Chlorogenic Acids: Recovery Yield from SCG and Content in Polar Molecules Fraction

A mass of 5 mg of the dry fraction of polar molecules was solubilized into 5 mL of hydroalcoholic solution EtOH/H_2_O (40/60, *v*/*v*). The resulting solution was filtered on syringe filter with a 0.22 μm PolyEtherSulfone (PES) membrane before HPLC-DAD analysis.

HPLC-DAD protocol for the analysis of polar molecules fraction, which is rich in chlorogenic acids and caffeine, was adapted from literature [55]. The full protocol is described in Supplementary Information (See Supplementary File).

## 3. Results

### 3.1. Study and Optimization of Lipids Extraction

#### 3.1.1. Description of Central Composite Rotatable Design Model Including ANOVA, Polynomial Equation, and Response Surfaces for Recovery Yield of Lipids

In this work, five levels of factorial design with three variables, pressure (bars), temperature (°C), and moisture content (wt%), were used for the design of experiments (DoE, Table 1). In order to establish the statistical significance of the studied parameters, predicted values and ANOVA tests of RYL were carried out (Table 2 and Table S1). Statistical analyses are fundamental to measure the impact of variation of parameters on the linear and two-way interactions between the factors. The experimental results of the design matrix were used to define the regression equation model describing the behavior of RYL at coded and un-coded levels (Table 2).

The response variable YRYL is presented as a polynomial second order function of simultaneous variations of the studied parameters. Positive and negative signs of the coefficients show that the variation of parameters is either agonistic or antagonistic to the final recovery yield of lipids [56]. The model’s coefficient R^2^ and the adjusted determination coefficient R^2^_adjusted_ for RYL are found to be statistically significant at 92.25% and 90.26%, respectively, which confirms the good suitability of the theoretical model to the experimental results [36].

The mathematical equation with values in coded levels is used for the determination of predicted RYL, as described in Supplementary Information (Table S1). These values are compared to the experimental ones and the predicted RYL value, compared to the value obtained experimentally are in good agreement. Deviations between measured and predicted RYL are calculated according to the following equation (Equation (6)):

Equation (6). Deviation between measured and predicted RYL

RYL Deviation = RYL_measured_ − RYL_predicted_ (6)

The lowest RYL deviation of −0.11 wt% was observed for experiment no. 7 corresponding to a pressure of 200 bars, a temperature of 50 °C, and a moisture contain of 40 wt% (0, 0, 0). The highest deviation of 16.53 wt% was noted for the experiment no. 11 corresponding to a pressure of 200 bars, a temperature of 66.8 °C, and a moisture content of 40 wt% (0, +α, 0). Those observations are confirmed by the normal probability plot for RYL, as observed in Supplementary Information (Figure S2).

The probability of each coefficient to be significant in the polynomial regression equation of RYL is established in the ANOVA test (Table 3). The adjusted sum squares (Adj SS) are very distinguishable, suggesting the major significance of certain parameters in favor to others. In addition, the variance distribution (F) has to be compared to the probability of the studied parameters. More precisely, the coefficients that present high Fischer’s test coefficients (F-value) combined with low probability (*p*-value) designate an important significance in the regression model [37]. For linear factors, the combination with the highest F-value and lowest *p*-value are reported for pressure. Contrarily, the combination of low F-value and high *p*-value is attributed to the moisture content factor, expressing its non-significance to influence the RYL response with 95% level confidence. For square and two-way interactions, the F-values, *p*-values couple is non-significant in general, with the exceptions of P2 and P*T. The F-value of P*T is reported to be superior to P2 or T, suggesting a strong synergistic interaction between pressure and temperature on the RYL response.

Polynomial regression equation is interpreted to give the weighting of the parameter and the type of influence, positive or negative, of those parameters on the RYL response (Table 2). Based on the equation in coded values, the pressure term P is considered as the most influent parameter. The P term is more than 2.5 times more influent than temperature T and more than 9 times more influent than moisture content M, in the limits of the studied area. Indeed, the influence of moisture content M is negligible on the RYL (*p* < 0.05). Due to its positive sign, the elevation of the pressure is related to an increase of the RYL response, whereas opposite trends are observed for temperature and moisture content. Like to ANOVA, P*T term is higher than T term. This observation suggests that the combination of specific pressure and temperature couple has a synergy that goes beyond the influence of temperature alone.

**Figure 1:**
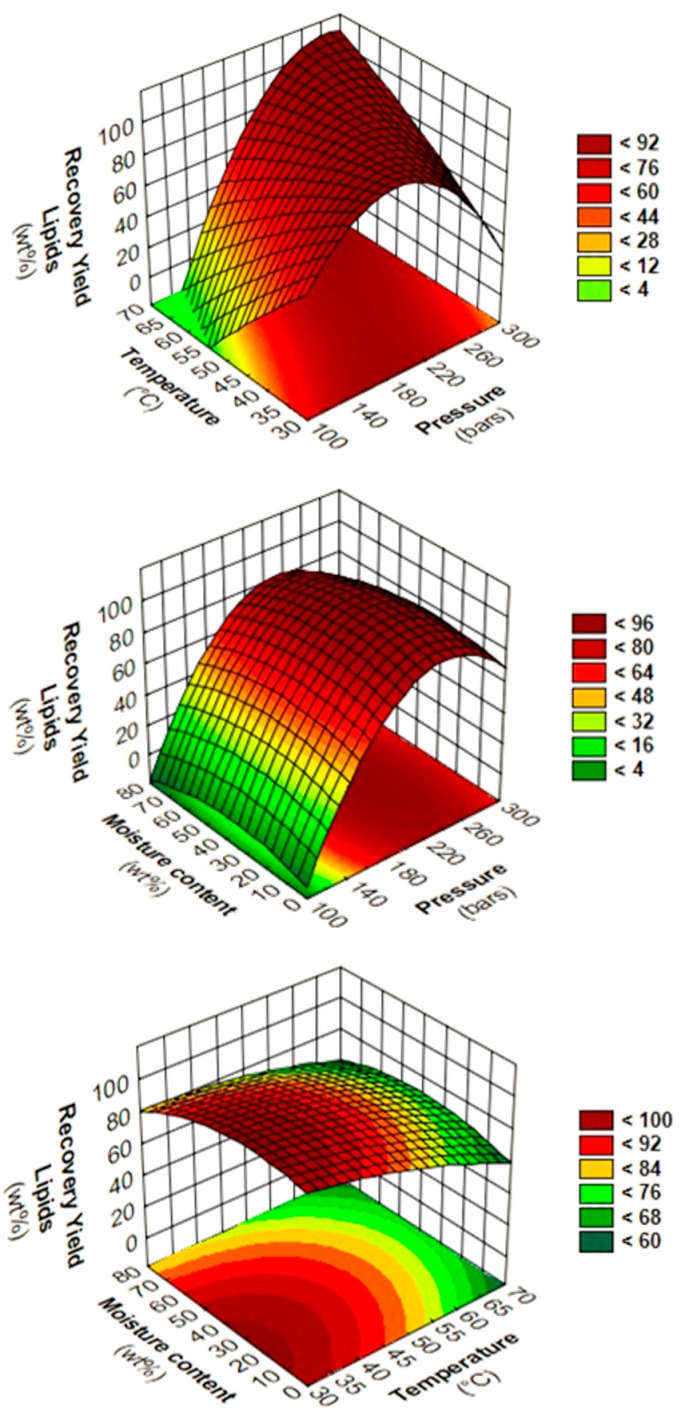
Response surfaces and contour plots of two-way interactions between pressure/temperature (**top**) at 40 wt% moisture content, pressure/moisture content at 50 °C (**middle**) and temperature/moisture content at 200 bars (**bottom**) for RYL of SC-CO_2_ extraction from SCG.

Contour plots and response surfaces are interpreted in order to accurately identify the influence of two-way interactions (Figure 1). The diagrams of two-way interactions between pressure and temperature at 40 wt% moisture content clearly show the bad influence to combine low pressure and high temperature. In another hand, a diagonal area that starts from 200 bars, 33.2 °C to 284.1 bars, and 66.8 °C was observed to give higher RYL. No significant trend was reported for the two-way interactions of P*M and T*M.

#### 3.1.2. Optimization of Recovery Yield of Lipids Using Desirability Function

The Design of Experiments based on Central Composite Rotatable Design were used to generate second order polynomial equation, which can be exploited to define operative parameters in order to target or minimize or maximize response variable. The optimized conditions for Recovery Yield of Lipids by SC-CO_2_ extraction of spent coffee grounds were assessed with the software Minitab V17 (Table 4). The optimal experiment to maximize RYL was performed in the following experimental conditions: Pressure = 284.1 bars, Temperature = 66.8 °C, and Moisture content = 6.4 wt%. To ensure the validation of the model, the difference between measured and predicted RYL must be lower than 5% [37]. Next, the RYL for measured and predicted data was reported as 94.89 and 99.58 wt%, resulting in *p*-value = 0.0470 (*p* < 0.05). The optimized conditions using desirability function allow (i) to measure RYL that fits with the model and (ii) to obtain the highest RYL reported in this study.

#### 3.1.3. Composition and Properties of Lipids Fraction

The characterization of lipids fraction obtained through SC-CO_2_ extractions without moisture, to optimize RYL, RYP, RYL+RYP, and with n-hexane Soxhlet reference from dry SCG, were reported in Table 4. The composition and properties of the SCG oils obtained by SC-CO_2_ in this study are (i) compared to the current literature (Kaffe Bueno company, see Table 5) and (ii) studied as a function of pressure, temperature, and moisture content based on this Design of Experiments in a further article.

#### 3.1.4. Effect of the Process Parameters on the Recovery Yield of Lipids

For the first time, the pressure, temperature, and moisture content parameters were studied for supercritical CO_2_ extraction of high value molecules from SCG with high moisture content up to 73.6 wt%. Those results were compared to the current literature and interpreted to explain the phenomena that occurred inside the SC-CO_2_ + H_2_O system.

The pressure term alone is the most influent of the studied parameters in this study. This is in accordance with literature, since Couto et al. also found major influence of pressure during supercritical CO_2_ extraction of dry SCG, with evolution of the yield from 4.2 to 13.1 g_oil_·100 g^−1^_SCG_ at 50 °C at 150 and 200 bars, respectively [40]. Similar trends were reported for lipid extraction from SCG with pure SC-CO_2_ in the studied range by numerous authors [29,58,59,60,61]. The increase of yield by increasing pressure might be explained by CO_2_ density that increases, which leads to an enhance of solvating power of CO_2_ [40]. For isothermal and isohumidity experiments with exclusively variation of pressure from 115.9 to 200 bars, the CO_2_ density strongly increased (Figure 1). Thereby, the RYL increases from 12.51 to 90.37 wt% with an increase of CO_2_ density from 556.1 to 784.29 kg·m^−3^, respectively, measured thanks to the NIST Chemistry WebBook [62,63,64]. The same variation of pressure above 200 bars does not lead to further improvement of the RYL, since the RYL reaches a plateau, where the density of CO_2_ is sufficient to solve efficiently lipid molecules. Then, the RYL is strongly dependent on CO_2_ density between 75 and 150 bars, where CO_2_ density varies significantly in the pressure range.

Contrarily, the temperature term alone is not significantly influent in this study, since temperature is one of the most difficult parameters to interpret from the obtained results. The increase of temperature reduces the density of CO_2_, decreasing the solvating power of CO_2_ [61]. In another hand, the increase of temperature leads to an increase of the vapor pressure of solute, resulting in higher solubility of solutes in CO_2_ [40]. The increase of temperature also decreases the viscosity of CO_2_, thereby promoting the diffusion of CO_2_ through the SCG matrix. In this study, the positive effects of temperature are predominant in a pressure above 200 bars, where density remains sufficient to dissolve lipids. Hence, the temperature influence should be interpreted as a function of the pressure.

Indeed, in this study, high temperature above 50 °C shows strong antagonistic effects when it is combined with pressure below or equal to 150 bars. The experiments that were performed at 150 bars, 20 wt% moisture content at 40 °C (−1), and 60 °C (+1) display RYL of 85.23 and 29.13 wt%, respectively. Concomitantly, significant differences of CO_2_ density from 780.23 to 604.09 kg·m^−3^ were observed. This negative combination of low pressure and high temperature is a phenomenon called retrogradation [60,65], as shown on the response surfaces or contour plots in the grey area of P*T parameters interactions (Figure 1).

The moisture content is the less influent factor of all the studied parameters, slightly negatively affecting the RYL. The negative influence of moisture content might be explained by the role of water that acts as a barrier for diffusion of CO_2_ [66], but in a negligible degree in this study.

The comparison of co-solvents, such as water from our work to ethanol from literature for supercritical CO_2_ extraction of SCG, is difficult. In the literature, authors performed their extraction experiments with SC-CO_2_ + EtOH without any liquid/liquid extraction to separate raw extracts into lipophilic and hydrophilic fractions, contrary to our study [29,40,59,61]. This is common to observe higher extraction yield with SC-CO_2_ + EtOH than the reference with n-hexane Soxhlet. For example, Couto et al. extracted 19.4 and 18.3 g·100 g^−1^ SCG for SC-CO_2_/EtOH (93.5/6.5 wt%) and n-hexane Soxhlet reference, respectively [40]. Therefore, it is impossible to clearly identify the influence of EtOH co-solvent. Indeed, SC-CO_2_ + EtOH could extract (i) exclusively lipophilic compounds in higher amounts in comparison to the reference, (ii) the same amount of lipophilic compounds as the n-hexane Soxhlet reference with additional hydrophilic compounds, or (iii) a lower amount of lipophilic compounds than reference, but compensated by the high amount of hydrophilic compounds.

Barbosa et al. also showed response surfaces for the amount of EtOH (wt%) and pressure (bars) at 70 °C, where rising EtOH co-solvent proportion enhances the SC-CO_2_ extraction yield of SCG oil [61]. The authors ascribed those results to the EtOH, which increases the affinity of SC-CO_2_ to more polar compounds. Indeed, the amount of polar molecules fraction can significantly modify the mass of SC-CO_2_ extract since polar molecules fraction represents up to 10.40 g_polar molecules_·100 g^−1^_SCG_ based on our hydroalcoholic extraction reference of defatted SCG. Thus, the polar molecules fraction represents a similar amount of matter than the lipids fraction up to 12.29 g lipids·100 g^−1^_SCG_, based on our n-hexane Soxhlet reference. To conclude, we strongly recommend separating SC-CO_2_ raw extract into lipophilic and hydrophilic fractions to have a better understanding of the role of co-solvent on the supercritical CO_2_ extraction of spent coffee grounds.

### 3.2. Study and Optimization of Polar Molecules Extraction

#### 3.2.1. Description of the Central Composite Rotatable Design Model Including ANOVA, Polynomial Equation, and Response Surfaces for Recovery Yield of Polar Molecules

In order to properly compare the extraction of polar molecules, the same design of experiments was used with a different response variable: Recovery yield of Polar molecules (RYP). The statistical significance of the studied parameters, predicted values, and ANOVA tests for RYP are reported in Table 6 and Table S1. The experimental results from the design matrix were used to define the regression equation model describing RYP behavior in coded and non-coded levels (Table 7).

The model’s coefficient R^2^ and the adjusted determination coefficient R2 adjusted for RYP are moderately significant at 71.25% and 63.86%, respectively, which is lower than the ones of RYL. The results suggest that the RYP model might be not as robust than the RYL model.

The mathematical equation with values in coded levels is used for the determination of predicted RYP, as described in Supplementary Information (Table S1). These values are given in comparison to the experimental ones. The lowest RYP deviation 0.02 wt% is observed for experiment no. 9 corresponding to the following parameters: P = 250 bars, T = 60 °C, moisture content = 20 wt% (+1, +1, −1). The highest deviation −1.54 wt% is noted for the experiment no. 38, corresponding to the following parameters: P = 200 bars, T = 66.8 °C, moisture content = 40 wt% (0, 0, −α). Those observations are confirmed by the normal probability plot for RYP, as observed in Supplementary Information (Figure S3).

The probability of each coefficient to be significant in the polynomial regression equation of RYP is established in ANOVA test (Table 6). For linear factors, the moisture content is reported to be the most influent parameter to modify the RYP response. For two-way interactions, no combination was significantly influent on the RYP response with 95% level confidence. The P*M and T*M are more significant than their linear factors P and T, significantly affecting the RYP response with 90% level confidence. The results for P*M and T*M interactions suggest a potential synergistic interaction between pressure or temperature with moisture content on the RYP response.

Based on the equation in coded values, the moisture content term M (0.830) is considered as the most influent parameter of all linear effects, which is more than 3.5 more influent than pressure P and more than 15.5 times more influent than temperature T terms (Table 7). For linear effects, the signs of P, T, and M terms in the equation are positive, showing an increase of the RYP response with the elevation of P, T, and M terms. P*M and T*M terms are higher than P and T terms alone, which show that that moisture content is a precondition in this study.

**Figure 2:**
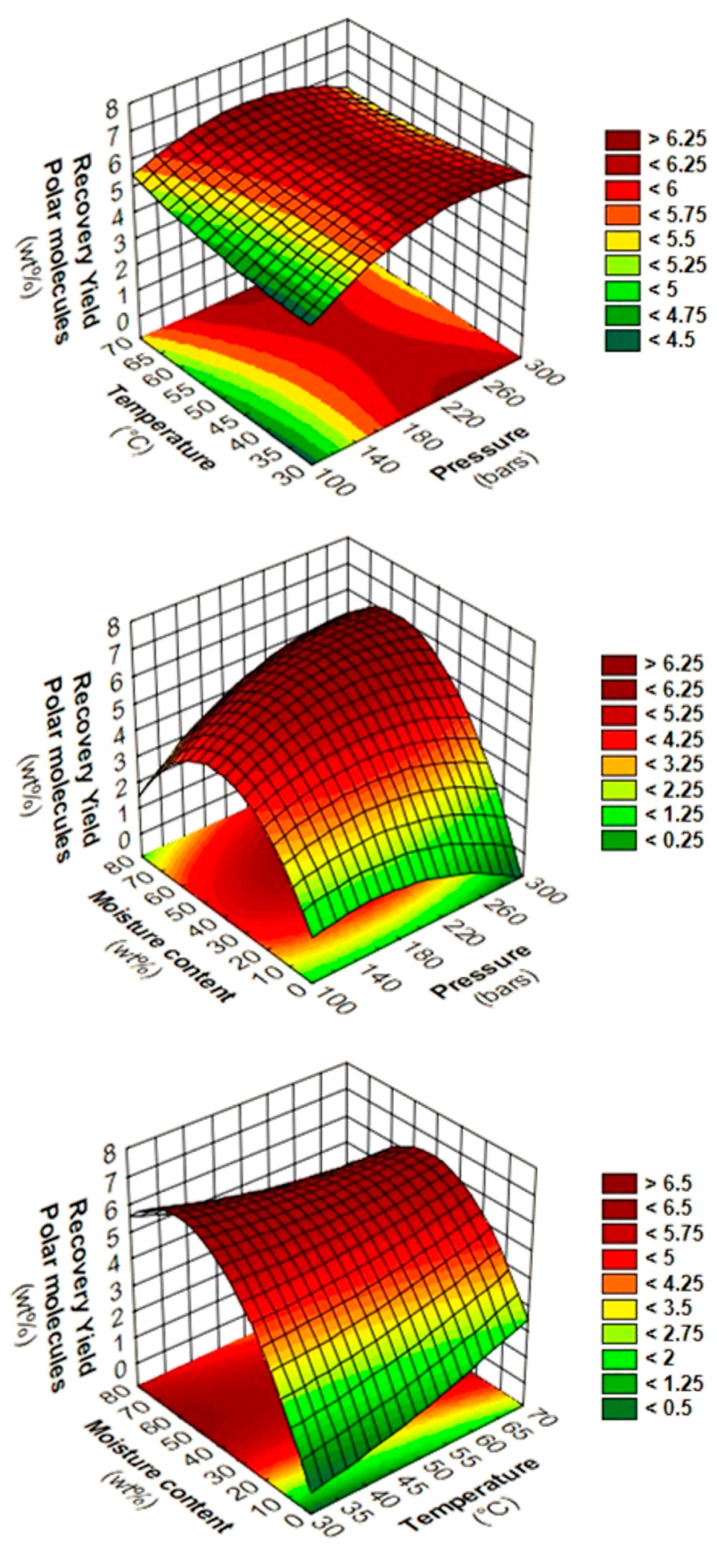
Response surfaces and contour plots of two-way interactions between pressure/temperature (**top**) at 40 wt% moisture content, pressure/moisture content at 50 °C (**middle**), and temperature/moisture content at 200 bars (**bottom**) for RYP of SC-CO_2_ extraction from SCG.

Contour plots and response surfaces are interpreted in order to reveal precisely the influence of two-way interactions (Figure 2). The diagrams of two-way interactions between pressure and temperature at 40 wt% moisture content show the weak influence of pressure and temperature, as observed in ANOVA and second-order polynomial equation. On another hand, diagrams of P*M at 50 °C and T*M at 200 bars reveal the same trends with optimum areas for RYP obtained between 40–60wt% moisture content.

#### 3.2.2. Optimization of Recovery Yield of Polar Molecules Using Desirability Function

The optimized conditions for Recovery Yield of Polar molecules by SC-CO_2_ extraction of spent coffee grounds were assessed with the software Minitab V17 (Table 4). The optimal experiment to maximize RYP was performed in the following experimental conditions: 270 bars, 40 °C and 60 wt% moisture content. The RYP for measured and predicted data are reported as 5.50 and 6.73 wt%, resulting in *p*-value: 0.2247 (*p* > 0.05, Table 4). The results from optimized conditions using desirability function are not acceptable, as the model does not fit with the reality with level confidence of 95%. Several hypotheses based on the effects of process parameters are pointed out, such as the important excess of water of 70 wt% of initial water mass due to low temperature (40 °C) and high moisture content (60 wt%) combination. The liquid/liquid extraction step might also influence the final result, since it adds extra steps with slightly different polar solvent depending on the amount of water extracted during the SC-CO_2_ extraction.

#### 3.2.3. Composition of Polar Molecules Fraction

Spent coffee grounds are rich in high-added value molecules such as caffeine and polyphenols such as free hydroxycinnamic acids or esterified as chlorogenic acid [67,68]. However, the amount of those high-added value molecules depends on several factors like the botanical specie of coffee, the geographical origin, the roasting process, or even the type of brewing [31].

Hence, polyphenols composition of SCG was determined in this study via a reference extraction method using an hydroalcoholic mixture EtOH/H_2_O (40/60 *v*/*v*) in order to extract nearly all the polyphenols in the SCG material according to the results published in the literature [31,69]. Characterizations of polar molecules fraction obtained by SC-CO_2_ extractions are reported in Table 8. An additional SCG extract prepared by conventional extraction with pure water without SC-CO_2_ was characterized and is presented in Table 8.

The SCG extract obtained by conventional hydroalcoholic extraction represent up to 10.40 wt% of the initial dry SCG (Table S1), with caffeine, 3-caffeoylquinic acid (3-CQA), and Total Hydroxycinnamic Acids free and bonded (THA) up to 8.33, 2.69, 9.54 g·100 g^−1^_extract_, respectively (Table 8). Thus, the composition of polar molecules fraction of the SCG exhibits caffeine, 3-CQA, and THA up to 8.66, 2.80, and 9.92 mg·g − 1SCG, respectively.

The amount of polar molecules fraction obtained from SC-CO_2_ extraction with dry SCG is extremely low since the dry extract of polar molecules, caffeine, and THA represents 0.021 g·100 g^−1^_SCG_, 0.04 mg·g^−1^_SCG_, and 0.06 mg·g^−1^_SCG_, respectively. In addition, the selectivity for caffeine extraction with pure SC-CO_2_ is weak, with caffeine up to 30.63 g·100 g^−1^_extract_.

On the contrary, the polar molecules fraction obtained from SC-CO_2_ extraction to optimize RYP or RYL + RYP is composed of caffeine of high purity, higher than 99 wt%. In addition, the recovery yield of caffeine is up to 65.97 wt%, compared to the extraction with hydroalcoholic reference (Table 8). Under the experimental conditions used to optimize RYP, the amount of THA is negligible in extract with 0.06 g·100 g^−1^_SCG_, despite the addition of H_2_O as a second more polar solvent. This raises questions about the ability of water to improve the extraction of hydroxycinnamic acids with SC-CO_2_.

However, SCG extract obtained by conventional extraction with pure water is composed of 8.22 mg·g^−1^_SCG_ of caffeine and 8.82 mg·g^−1^_SCG_ of THA. Those results show the high efficiency of water to recover polyphenols since the caffeine and THA recovery yield are up to 94.92 and 88.89 wt%, respectively, compared to the extraction with hydroalcoholic reference.

#### 3.2.4. Effect of the Process Parameters in SC-CO_2_/H_2_O/SCG System for Caffeine Extraction

The water in SC-CO_2_ shows strong limitations concerning recovering polar molecules such as polyphenols, since the highest yield reported for RYP with SC-CO_2_ + H_2_O is 6.74 wt%. It represents a weak yield of 0.70 g_polar.molecules_·100 g^−1^_SCG_ in comparison with the 10.40 g_polar.molecules_·100 g^−1^_SCG_ was obtained with the reference hydroalcoholic extraction (Table S1). This limitation might be due to the apolar properties of CO_2_, which is not modified by CO_2_ saturated in H_2_O poorly up to 0.15–0.30 g_H2O_·100 g^−1^_CO2_, depending of the pressure/temperature/moisture content combination. Since solid/liquid extraction of SCG pure water allows to recover 86.53 wt% of polar molecules from SCG (9.00 g_polar.molecules_·100 g^−1^_SCG_), it can be deduced that water does not significantly modify the polarity of SC-CO_2_ due to an extremely low yield of SC-CO_2_ + H_2_O compared to H_2_O alone.

Tello et al. have also studied the influence of natural humidity between 16–64 wt% in coffee husks for the SC-CO_2_ extraction of caffeine [70]. The authors reported (i) the very low amount of caffeine extracted at low moisture content (16.4 wt%), (ii) the optimum amount of caffeine extracted with great efficiency of medium moisture content (32 and 48 wt%), and (iii) the significant drop of caffeine extracted at higher moisture contents (64 wt%) [70]. Authors suggested that water favors the hydrolytic rupture of hydrogen bonds between caffeine and the natural matrix [70,71]. This hypothesis is supported by the fact that water is a naturally good solvent to extract caffeine or polyphenols from SCG [31]. In addition, water might help the swelling of the cell membrane, which leads to the enhancement of solute diffusion [70,72,73]. However, this hypothesis is not prevalent for spent coffee grounds since it is coffee that was grinded into fine powder, which increases the specific surface, thus enhancing solute diffusion without the need for water. Finally, Iwai et al. noticed that SC-CO_2_ with saturated water leads to a 22% increase in the solubility of caffeine at 150 bars, 40 °C through the modification of the polarity of SC-CO_2_ in these conditions [74].

In this study, the temperature has a negligible influence compared to moisture content since it is a precondition to the suitable extraction of caffeine. However, the increase of temperature leads to a slight increase to extract caffeine that might be due to the decrease of viscosity of CO_2_ which promote the solute diffusion (Figure 2). Indeed, Menzio et al. reported the influence of temperature for the SC-CO_2_ extraction of caffeine from wet coffee beans with a constant moisture content of 31 wt% during 1 h [75]. The authors observed that increasing temperature from 40 to 75 °C increased the yield by 2.5 times from 32.0 to 83.8 mg_caffeine_·100 g^−1^_coffee_, respectively.

In this study, the pressure also had a negligible influence compared to moisture content. However, the predicted yield of caffeine can almost double from 115.9 to 284.1 bars at a high moisture content of 73.6 wt%. This might be due to the density of CO_2_ that affects the poor solubility of H_2_O, which is around 0.3 wt%, and the high pressure of CO_2_ could lead to higher water co-extraction in our dynamic SC-CO_2_ system [73]. Depending on initial moisture content and pressure, the excess of water that remains in the extractor at the end of extraction is very important, since the solute will remain in this excess of water instead of being extracted in SC-CO_2_ [70,73].

Water is thus a unique co-solvent that allows for recovering more than 65 wt% of caffeine very selectively among the polar molecules (>99 wt% purity), contrary to EtOH. Effectively, Araujo et al. reported very a low concentration of caffeine in extracts by SC-CO_2_ experiments at 100–200 bars, 40–80 °C, with or without 50–200 g_EtOH_·g^−1^_SCG_ co-solvent from 0.064 to 0.712 g_CAF_·100 g^−1^_extract_. Most of the raw extract is composed of lipids, whereas polar molecules compounds represent a minor part up to 0.064–0.711 g·100 g^-1^_oil_ for caffeine and 0.00397–0.00936 g·100 g^−1^_oil_ for caffeic acids. By comparison, these results are way inferior to the 5.72 mg_CAF_·g^−1^_SCG_ obtained under RYP optimized condition at 270 bars, 40 °C, 60 wt% moisture content presented in the current work, despite different chemical composition inherent to the starting raw material. In addition, Araujo et al. exhibited that hydroxycinnamic acids such as caffeic, *p*-coumaric, and ferulic acids are ten times less extracted than caffeine with SC-CO_2_ + EtOH and not detected with pure SC-CO_2_ [29]. Based on those observations, EtOH is an appropriate co-solvent to enrich oil in high value molecules such as caffeine and hydroxycinnamic acids compared to pure CO_2_, but remains insufficient to exhaust the raw material in those molecules. This is confirmed in the same study by the lack of improvement by using pressurized EtOH, which leads to a concentration of caffeine in SCG of 0.209 to 0.682 mg_CAF_·g^−1^_SCG_ [29]. Indeed, it is confirmed in the literature that the use of pure EtOH at standard pressure leads to a sharp drop in caffeine extracted [31] due to the inability of EtOH to break down the linkages between caffeine and/or polyphenols and the matrix.

#### 3.2.5. Mechanism of Caffeine Extraction in SC-CO_2_/H_2_O/SCG System

The necessity of additional solvent during supercritical CO_2_ extraction of caffeine from SCG is questionable when it is known that pure caffeine is easily soluble in pure CO_2_ at 40 °C between 100 to 300 bars up to 6.3 to 37 × 10^−5^ mole fraction, respectively [76]. Indeed, in this study, experiments were performed using 3000 g_CO2_ or 3 kg_CO2_ per experiment, which could potentially solubilize 1.63 g_caffeine_·kg^−1^_CO2_ or 4.89 g_caffeine_ with 3 kg of CO_2_. However, 0.21 g of caffeine was extracted from the 25 g of SCG in our experiments, since caffeine might interact and be retained into the vegetable matrix.

**Figure 3:**
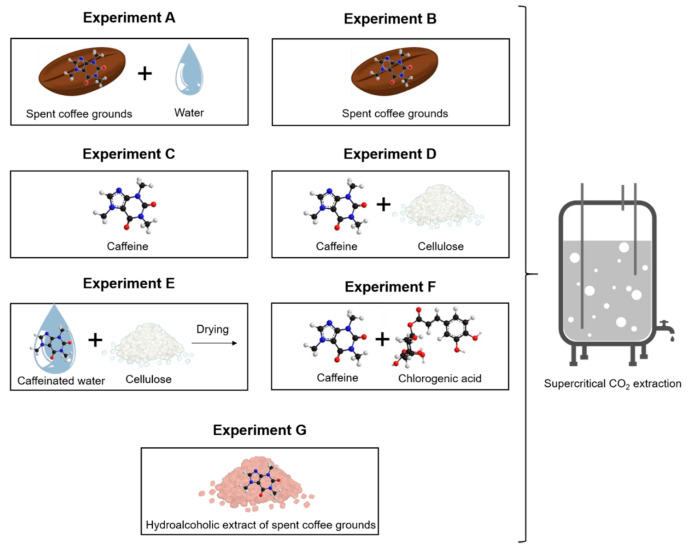
Explanatory diagram of additional manipulations performed with supercritical CO_2_ at 265 bars and 55 °C to understand the mechanism of extraction with supercritical CO_2_ in the presence of water.

**Figure 4:**
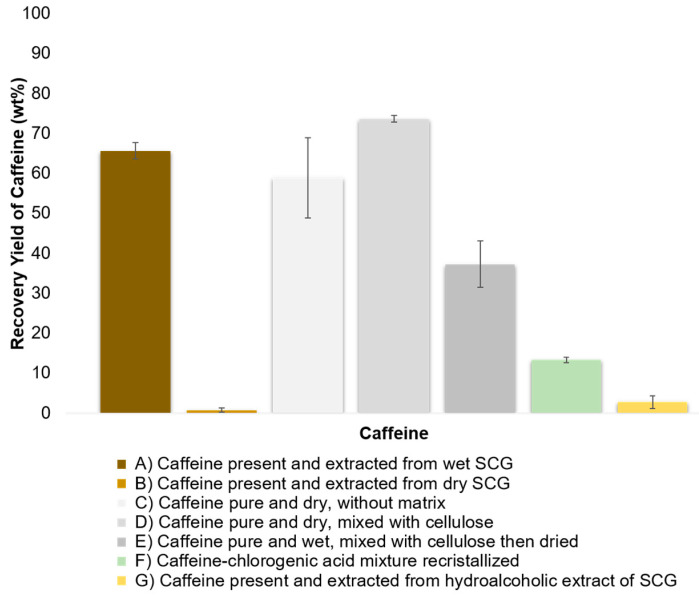
Recovery yield of caffeine extracted from different matrices by supercritical CO_2_ extractions.

Industrial decaffeination processes of green coffee beans are intended to operate by soaking beans with water before extraction with organic solvent or SC-CO_2_ [77]. Authors of works with SC-CO_2_/H_2_O/SCG or SC-CO_2_/H_2_O/GCB systems proposed one or more hypotheses that can work together or separately to define the role of water in the mechanisms of caffeine extraction [70,71,72,73,74,78,79,80,81]. The water might (i) lead to the hydrolytic rupture of hydrogen bonds between adsorbed caffeine to the natural matrix, before being dragged by supercritical CO_2_, (ii) contribute to the swelling of cell membrane favoring solute diffusion, or (iii) be dissolved into SC-CO_2_. In order to highlight the prevalent role of water, additional experiments were conducted, as schematized in Figure 3, and the results are presented in Figure 4.

The experiments (A) and (B) expose the large influence of water for the caffeine extraction from SCG.

The experiments (C), (D), and (E) were conducted with pure caffeine. The reason for mixing caffeine into cellulose without adding water is to more homogeneously caffeine disperse inside the aluminium basket extraction, which increases the contact surface with supercritical CO_2_. As expected, more caffeine was extracted in experiment (D) (73.63 wt%) than in experiment (C) (58.95 wt%), since caffeine is more accessible for supercritical CO_2_, which results in a higher amount of g_CO2_·g^−1^_CAF_. In addition, it was noticed that the amount of caffeine extracted from experiment (C) and (D) is similar to the amount of caffeine extracted from wet SCG.

The experiment (E) was prepared by mixing a water solution of caffeine to spread caffeine homogeneously on the cellulose before being dried. Contrary to the experiment (D), in experiment (E) the soaking of SCG with H_2_O/caffeine mixture, followed by the drying step, can lead to significant interactions such as Van Der Waals interactions and hydrogen bonds that retain the caffeine to the matrix. Kobetičová et al. studied the interaction of caffeine with the wood, including the study of each wood fraction like cellulose [82]. The authors clearly showed the absence of water solution of caffeine interaction with cellulose without drying. Thereby, the drying of aqueous caffeine lead to its impregnation into cellulose, which results in the high interactions observed in this study, which are not explained in Kobetičová et al. or in this study.

The experiment (F) was carried out to determine the solubility of caffeine when wa is mixed with a chlorogenic acid. Indeed, chlorogenic acid and caffeine form a complex through the intermolecular interaction of conjugated double bonds rather than hydrogen bonds [83]. In the present study, caffeine was recrystallized in the presence of 10%mol excess of chlorogenic acid. The supercritical CO_2_ extraction of the chlorogenic acid/caffeine complex results in a low recovery rate of caffeine of 13.34 wt%. This could confirm the hypothesis in which the caffeine and chlorogenic acid are strongly interacting in this mixture or in SCG. Therefore, SC-CO_2_ is not strong enough to break the interactions of the complex or to solubilize it due to the lack of chemical interactions between the solute and SC-CO_2_ solvent. Even if the caffeine in experiment (F) is not complexed to chlorogenic acid, which is an esterified hydroxycinnamic acid, Kobetičová et al. demonstrated that caffeine strongly interacts with hydroxycinnamic acids like coumaryl alcohol [82].

The experiment (G) was performed from a hydroalcoholic extract of SCG, which contains up to 8.66 g·100 g^−1^_extract_ of caffeine and 9.54 g·100 g^−1^_extract_ of total hydroxycinnamic acids, respectively. The experiment (G) shows a very low extraction yield of caffeine of 2.78 wt%, regardless of the easy physical accessibility for SC-CO_2_ to caffeine. Thereby, the water could have a role to expand and open the coffee cells matrix, but this role is not the prevalent one. Indeed, despite the grinding process, it might be possible that metabolites are trapped into cellular tissues, where water allows for its diffusion through the coffee matrix by swelling effects, as proposed by several authors [70,79]. However, caffeine presents in the dry hydroalcoholic extract that is separated from the coffee biological cells is not extractible by SC-CO_2_. Then, the prevalent role of water can be discriminate, which is not to open a physical path for SC-CO_2_ through swelling effects of coffee matrix.

**Figure 5:**
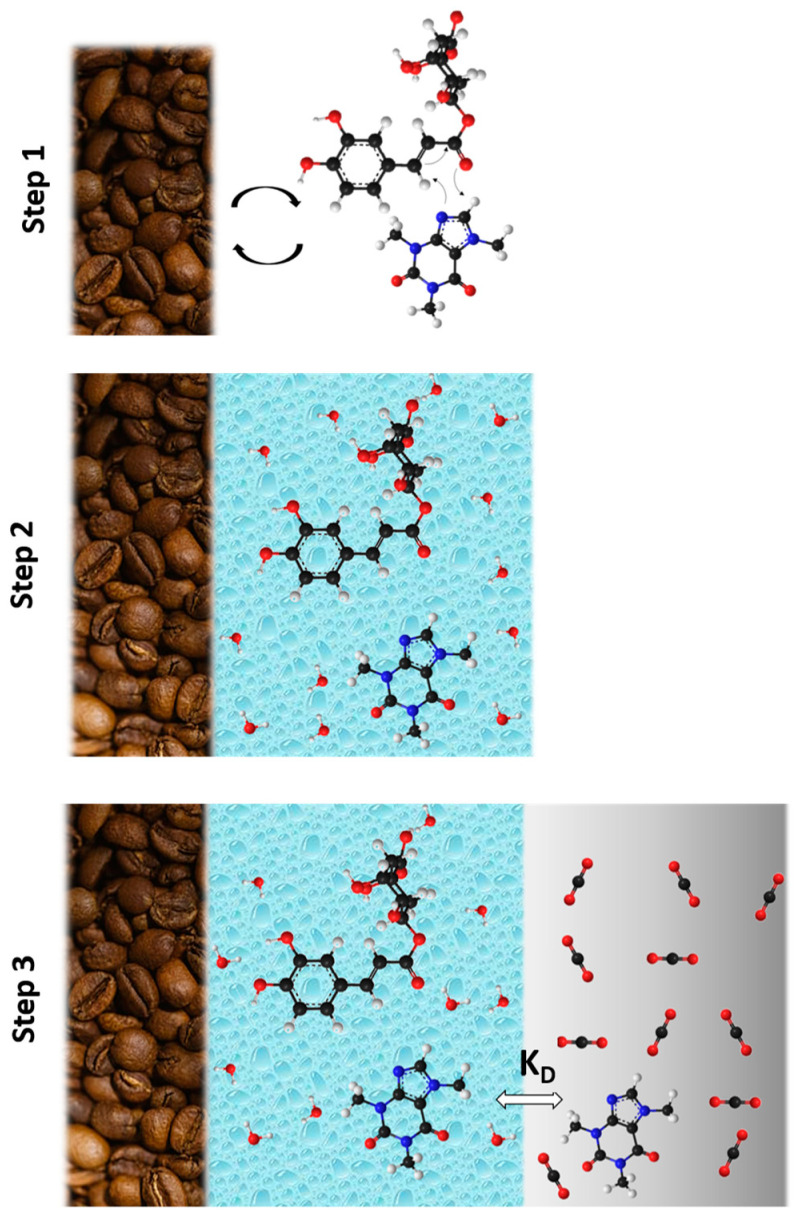
Mechanism of extraction of caffeine: step 1 --> no water, interactions between caffeine and chlorogenic acids and matrix, step 2 --> solubilization of caffeine in water, and step 3 --> SC-CO_2_/H_2_O liquid/liquid extraction of caffeine.

Based on the current state of art and results from this study, the prevalent mechanism during SC-CO_2_ + H_2_O extraction of caffeine from SCG is fully detailed (Figure 5).

The mechanism of extraction for caffeine from spent coffee grounds could be explained by the mechanism in two steps: (i) water breaks interactions of caffeine with chlorogenic acids and lignocellulosic matrix, and caffeine becomes a molecule at a free state and (ii) water enriched in polar molecules from coffee exchanges caffeine with the large excess of SC-CO_2_ of 218 g_CO2_·g^−1^_H2O_, which is highly selective with caffeine. This hypothesis is supported by several observations and results from this study.

Firstly, the kinetic of extraction of polar molecules by SC-CO_2_ follows a logarithmic curve when the curve of amount of water extracted is linear during the first 45 min. However, the amount of caffeine extracted is not proportional to the 0.3 wt% water solubilized into SC-CO_2._ Despite the increase of polarity of SC-CO_2_ when saturated in H_2_O, it remains insufficiently polar to extract chlorogenic acids, probably due to a very low amount of 0.3 wt% of water solubilized.

Secondly, based on results presented in Figure 4, the chlorogenic acid–caffeine complex might be one of the main reasons, since it naturally occurs in coffee where it forms a conjugated system, as demonstrated in literature [83,84,85]. Those interactions are showed to be very strong in a complex matrix system like spent coffee grounds, where the affinity between caffeine and SC-CO_2_ is insufficient.

Thirdly, the phenomenon of liquid/liquid extraction that might occurr in the system is supported by the observation of the response surfaces of RYP. Indeed, above a certain threshold of moisture content at 60 wt%, the RYP starts to fall off. This might suggest that the excess of water that remains in the SC-CO_2_/H_2_O system is more important at low moisture content, thus the equilibrium of caffeine switches slightly more to water. Otherwise, if water only breaks caffeine interactions, the excess of water would not lead to any increase or decrease of caffeine since it probably competes with SC-CO_2_ to solubilize caffeine, as observed in this study. More precisely, moisture content above 60 wt% leads to an excess of residual water undried in SCG which directly competed with SC-CO_2_ during liquid/liquid extraction, thus retaining more caffeine in the biomass matrix.

### 3.3. Study and Optimization of Lipids and Polar Molecules Simultaneous Extraction

#### 3.3.1. Optimization of Recovery Yield of Lipids and Recovery Yield of Polar Molecules Using Desirability Function

The desirability function (DF) or Derringer desirability function is used for simultaneous optimization of multiple responses of a process, suggesting levels of independent variables, providing the best balance among several different response variables. The DF is comprised between 0 and 1, with DF close to 1, which refers to experimental conditions designing a strong desirable limit [86]. This methodology facilitates the experimental analysis by converting a multiple response optimization problem into a single response that is easier to interpret [87,88]. The optimized conditions for Recovery Yield of Lipids and Recovery Yield of Polar molecules were assessed with Desirability Function (DF) in the software Minitab V17 (Table 4). Given the optimal experiment to simultaneously maximize RYL with DF of 1.0000 and RYP with DF of 0.9123, the RYL + RYP desirability function of 0.9551 was obtained in the following experimental conditions: 265 bars, 55 °C, and 55 wt% moisture content. The RYL for measured and predicted data are reported as 92.68 and 93.97 wt%, resulting in p-values of 0.0137 (*p* < 0.05). The RYP for measured and predicted data are 5.3591 and 6.2039 wt%, resulting in a *p*-value of 0.1362 (*p* > 0.05). The model fits better for RYL + RYP simultaneously but it is still not acceptable in level confidence of 95% for RYP. The optimization of RYL and RYP enables us to extract 11.39 g_oil_·100 g^−1^_SCG_ and 0.56 g_polar.molecules_·100 g^−1^_SCG_.

#### 3.3.2. Kinetic of Optimized Extraction for Recovery Yield of Lipids and Recovery Yield of Polar Molecules

**Figure 6:**
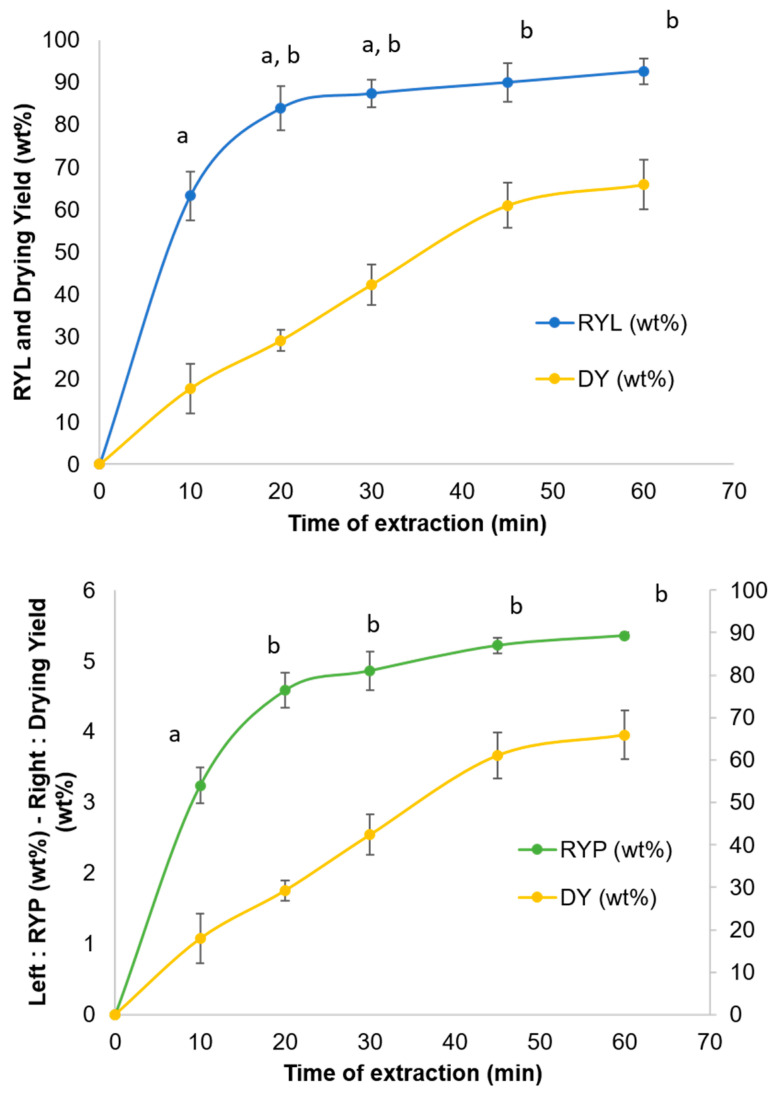
Extraction kinetic for RYL (**top**) and RYP (**bottom**) in optimized conditions (265 bars, 55 °C, 55 wt% moisture content). Values marked by the same letters are not significantly different according to the Student test (*p* < 0.05).

The kinetic of the optimized extraction is crucial for RYL and RYP to identify the plateau where the maximal yield is reached and to compare the evolution of the yield of extraction for lipids and caffeine with the evolution of water dried in order to verify if water acts as a barrier for solute diffusion in the early stages of extraction. The kinetics of the experiments were performed for one hour with the same time of extraction as in the experiments of the Design of Experiments, at 10, 20, 30, 45, and 60 min (Figure 6).

The RYL and RYP kinetic curves present a logarithmic appearance with strong rise of the extraction yield from 0 to 20 min, followed by a plateau where the maximum seems to be reached. On the contrary, the Drying Yield (DY, Equation (7)) evolves linearly from 0 to 45 min until it reaches a plateau at around 65 wt% of drying.

Equation (7). Drying Yield (DY)

(7) $$Drying\;Yield\;\left(DY\right)=\frac{m_{H_{2}O}\;Initial-m_{H_{2}O}\;Final}{m_{H_{2}O}\;Initial\;}$$

This absence of proportional relation between RYP and DY confirms that water acts before the SC-CO_2_ to unbind caffeine to the matrix. Otherwise, the CO_2_ saturated with water would extract the caffeine in a proportional way as a function of solubilized water in SC-CO_2_ from SCG.

Student tests or *t*-tests were carried out to statistically determine the minimum time of extraction, where no significant differences are observed for RYL and RYP at 60 min. The RYL at 10 min presents significant statistical differences to the RYL obtained at 45 and 60 min (*p* < 0.05). Otherwise, non-significant statistical differences are reported for the comparison of other RYLs between 20 and 60 min (*p* > 0.05). A similar trend is observed for RYP, with significant statistical differences between the RYP obtained at 10 min and the ones at 20, 30, 45, and 60 min (*p* < 0.05). Student tests for RYP show no significant statistical differences between 20 and 60 min (*p* > 0.05).

As a conclusion, no significant statistical differences according to the Student test (*p* > 0.05) were observed for RYL and RYP above 20 min of SCG SC-CO_2_ extraction. Therefore, 20 min of SC-CO_2_ extraction at 265 bars, 55 °C, and 55 wt% moisture content increases the productivity of SCG oil extraction by 3, cuts down power consumption by 3, and reduces CO_2_ used to 40 g_CO2_·g^−1^_SCG_ against 120 g_CO2_·g^−1^_SCG_, where 120 g_CO2_·g^−1^_SCG_ corresponds to the amount of CO_2_ used after 60 min of extraction.

## 4. Conclusions

Supercritical CO_2_ under optimized conditions, i.e., P = 265 bars, T = 55 °C, and moisture content = 55 wt%, was successfully used to simultaneously recover up to 92.67 wt% of lipids and 5.36 wt% of caffeine.

The moisture content in the SCG has no significant influence on the recovery of lipids, thus, water does not act as barrier to CO_2_ diffusion. Contrarily, pressure is the most influent parameter due to its influence on the density of CO_2_, and thus to the CO_2_ solvation power of lipids.

The water in the SCG is a key factor in recovering the caffeine. The prevalent role and mechanisms of water during SC-CO_2_ + H_2_O extraction of caffeine from SCG are detailed and highlighted. Water acts as an immiscible solvent which modifies the molecular state of caffeine in matrix, contrary to EtOH co-solvent that induces a change in the polarity of supercritical CO_2_. Thus, water breaks caffeine interactions with chlorogenic acids before selectively transferring caffeine into SC-CO_2_ by liquid/liquid extraction.

The results obtained in this study offer many perspectives to exploit. Secondly, the energy enthalpies of the transition from chlorogenic acid–caffeine complex, for which solvate molecules inside SC-CO_2_/H_2_O system are not yet calculated. The modelling of this particular system using Density Functional Theory (DFT) could enlighten knowledge about interactions of this trilateral agreement between SC-CO_2_/H_2_O/CAF_SCG_. The DFT already proved its worth to get a better comprehension of binding energies of molecules during biomass extraction, biomass transformation, or in a supercritical system like supercritical H_2_O [89,90,91]. Hence, it could be useful to have a better understanding of the SC-CO_2_/H_2_O system to apply it to other biomasses.

Thirdly, this work exposes that the SC-CO_2_/H_2_O system is a unique process to recover the caffeine from SCG with a high selectivity. More precisely, molecular species in the SCG matrix could be defined as extractible in pure SC-CO_2_ like lipids or extractible in SC-CO_2_ pure, but that interacts strongly with its natural matrix, which required the assistance of water like caffeine and/or extractible in water or hydroalcoholic solvents like caffeine and chlorogenic acids. The use of these three extractions successively could allow for recovering high value molecules present in hydroalcoholic extract as pure or highly enriched species after the second step from other biomasses. The advantages of the supercritical CO_2_ and H_2_O system could be applied to other biomasses with methylxanthine and polyphenols content, such as cocoa pods [92,93,94].

Finally, this work shows a clear phenomenon of liquid/liquid extraction between SC-CO_2_ and H_2_O during the supercritical fluid extraction of spent coffee grounds. These results promote the development of green solvents immiscible to SC-CO_2_, which might first break solutes interactions to its matrix. Then, the solute present in these green solvents could be able to be selectively transferred into SC-CO_2_ by liquid/liquid extraction. For example, Ionic Liquids (IL) such as [C_4_C_1_im][PF_6_] and Deep Eutectic Solvent (DES) such as choline chloride–urea are innovative solvents that are not miscible in SC-CO_2_, which have rarely been investigated yet for biomasses valorization combined with SC-CO_2_ as SC-CO_2_/IL/Biomass or SC-CO_2_/DES/Biomass systems [95,96,97].

## Figures and Tables

**Table 1:** Independent variables and their level used (α: 1.68) in central Composite Rotatable Design (CCRD) for the optimization of supercritical CO_2_ extraction of SCG.

Independent Variables	Unit	Level of the Factors				
		−α	−1	0	+1	+α
Pressure	bars	115.9	150	200	250	284.1
Temperature	°C	33.2	40	50	60	66.8
Moisture content	wt%	6.4	20	40	60	73.6

**Table 2:** Second order polynomial equations for the response of RYL for SC-CO_2_ extraction of SCG.

	Recovery Yield of Lipids (RYL)	
Type of Units	Second-Order Polynomial Equation ^a,b^	R^2^
Coded ^c^	Y_RYL_ = 90.47 + **19.19** P − 7.43 T − 2.08 M − 13.82 P*P − 1.30 T*T − 2.75 M*M + 13.72 P*T − 0.33 P*M + 1.18 T*M	0.9225
Non-coded ^d^	Y_RYL_ = 74.0 + 1.236 P − 5.17 T + 0.217 M − 0.005526 P*P - 0.0130 T*T − 0.00689 M*M + 0.02743 P*T − 0.00033 P*M + 0.00591 T*M	

^a^ The letters represent the different independent variables: P for Pressure (bars), T for Temperature (°C) and M for Moisture content (%wt); ^b^ The bold parameter is the most significant one according to the second order polynomial equation; ^c^ The values of the coded levels are included between −α < P, T, M < +α with α = 1.68; ^d^ The values of the non-coded levels are included between 115.9 < P < 284.1 bars, 33.2 < T < 66.8 °C, 6.4 < M < 73.6 wt%.

**Table 3:** ANOVA analysis of Recovery Yield of Lipids for SC-CO_2_ extraction of SCG.

Source		Recovery Yield of Lipids (RYL)				
		DF	Adj SS	Adj MS	F-Value	*p*-Value *
Model		9	27,444.4	3049.4	46.29	<0.001
Linear		3	17,532.5	5844.2	88.72	**<0.001**
	P	1	15,094.1	15,094.1	229.15	**<0.001**
	T	1	2261.5	2261.5	34.33	**<0.001**
	M	1	176.9	176.9	2.69	0.110
Square		3	5359.9	1786.6	27.12	**<0.001**
	P^2^	1	3466.1	3466.1	52.62	**<0.001**
	T^2^	1	30.6	30.6	0.46	0.500
	M^2^	1	137.8	137.8	2.09	0.157
Two-way interactions		3	4551.9	1517.3	23.04	**<0.001**
	P*T	1	4515.8	4515.8	68.56	**<0.001**
	P*M	1	2.6	2.6	0.04	0.845
	T*M	1	33.6	33.6	0.51	0.480
Error		35	2305.4	65.9		
Lack of Fit		5	2177.0	435.4	101.75	**<0.001**
Total		44	29,749.8			

* *p*-values in bold represent parameters that are statistically significant in the CCRD (*p* < 0.05).

**Table 4:** Optimization of RYL, RYP, and RYL/RYP simultaneously with Derringer’s desirability function (DF).

Experience	Optimized RYL	Optimized RYP	Optimized RYL + RYP	
Response Variable	RYL	RYP	RYL	RYP
Pressure (bars)	284.1	270.0	265.0	
Temperature (°C)	66.8	40.0	55.0	
Moisture content (wt%)	6.4	60.0	55.0	
Desirability Function	1.0000	0.9991	1.0000	0.9123
			0.9551	
Predicted Response	99.58	6.73	93.97	6.20
Measured Response	94.89	5.50	92.68	5.36
*p*-value	0.04709 (*p* < 0.05)	0.2247 (*p* > 0.05)	0.0137 (*p* < 0.05)	0.1362 (*p* > 0.05)

**Table 5:** Characterization of lipids fractions by GC-MS for Fatty Acids Profile (FAP) and Fatty Acids Level (FAL), by colorimetric titration for Acid Value (AV), Saponification Value (SV), Ester Value (EV), and Iodine Value (IV), by physico-chemical analyses for Density, Dynamic Viscosity, Kinematic Viscosity, Refractive Index, and UV-Visible spectrophotometer for Trolox Equivalent Antioxidant Capacity (TEAC).

Experiments		n-Hexane Soxhlet (C_6_H_14_)	RYL Optimization (SC-CO_2_ + H_2_O)	RYP Optimization (SC-CO_2_ + H_2_O)	RYL + RYP Optimization (SC-CO_2_ + H_2_O)	No Water (SC-CO_2_)	Kaffe Bueno Oil (SC-CO_2_) [57]
Fatty Acids Profile (wt%)	C16:0	30.25	34.78	36.08	39.24	36.58	34
	C18:0	7.41	7.07	6.98	6.61	6.98	7.3
	C18:1	10.58	10.02	9.71	9.30	9.73	9
	C18:2	48.87	45.34	44.49	42.30	43.95	44
	C20:0	2.89	2.79	2.74	2.55	2.77	2.6
Fatty Acids Level (wt%)		42.92	49.81	47.51	53.37	46.68	/
Acid Value (mg_KOH_·g^−1^_oil_)		12.88	10.41	12.07	14.73	13.32	2–6
Saponification Value (mg_KOH_·g^−1^_oil_)		173.65	171.37	171.61	172.56	167.34	194
Ester Value (mg_KOH_·g^−1^_oil_)		160.77	160.96	159.54	157.83	154.02	188–192
% Free Fatty Acids		7.42	6.07	7.03	8.54	7.96	1–3
Iodine Value (g_I_·100 g^−1^_oil_)		77.41	67.04	69.12	72.80	72.96	90
Density ρ (kg·m^−3^)	20 °C	940.08	934.67	933.83	935.04	933.94	/
	40 °C	926.04	920.90	920.06	921.23	920.16	/
Dynamic Viscosity µ (mPa·s) at 40 °C		64.815	54.748	54.355	51.857	51.622	/
Kinematic Viscosity ν (mm^2^·s^−1^) at 40 °C		69.737	59.450	59.078	56.291	56.102	/
Refractive index n at 20 °C		1.47854	1.476667	1.477037	1.47758	1.477144	/
TEAC (µmol_TE_·100 g^−1^_oil_)		2319.1	359.3	437.2	700.2	709.4	/

**Table 6:** ANOVA analysis of Recovery Yield of Polar molecules for SC-CO_2_ extraction of SCG.

Source		Recovery Yield of Polar Molecules (RYP)				
		DF	Adj SS	Adj MS	F-Value	*p*-Value ^a^
Model		9	59.3878	6.5986	9.64	**<0.001**
Linear		3	30.2683	10.0894	14.74	**<0.001**
	P	1	1.9339	1.9339	2.82	0.102
	T	1	0.1134	0.1134	0.17	0.687
	M	1	28.2211	28.2211	41.22	**<0.001**
Square		3	24.0256	8.0085	11.70	**<0.001**
	P^2^	1	1.0840	1.0840	1.58	0.217
	T^2^	1	0.0794	0.0794	0.12	0.735
	M^2^	1	13.3089	13.3089	19.44	**<0.001**
Two-way interactions		3	5.0939	1.6980	2.48	0.077
	P*T	1	0.2832	0.2832	0.41	0.524
	P*M	1	2.7403	2.7403	4.00	0.053
	T*M	1	2.0703	2.0703	3.02	0.091
Error		35	23.9622	0.6846		
Lack of Fit		5	21.4969	4.2994	52.32	**<0.001**
Total		44	29749.8			

^a^ *p*-values in bold represent parameters that are statistically significant in the CCRD (*p* < 0.05).

**Table 7:** Second order polynomial equations for the response of RYP for SC-CO_2_ extraction of SCG.

Type of Units	Recovery Yield of Polar Molecules (RYP)	
	Second-Order Polynomial Equation ^a,b^	R^2^
Coded ^c^	Y_RYP_: 6.002 + 0.217 P + 0.053 T + **0.830 M** − 0.244 P*P + 0.066 T*T − 0.856 M*M − 0.109 P*T + 0.338 P*M − 0.294 T*M	0.7125
Non-coded ^d^	Y_RYP_: −4.88 + 0.0408 P + 0.041 T + 0.2186 M − 0.000098 P*P + 0.00066 T*T − 0.002140 M*M − 0.000217 P*T + 0.000338 P*M − 0.001469 T*M	

^a^ The letters represent the different independent variables: P for Pressure (bars), T for Temperature (°C), and M for Moisture content (%wt); ^b^ The bold parameter is the most significant one according to the second order polynomial equation; ^c^ The values of the coded levels are included between −α < P, T, M < +α with α = 1.68; ^d^ The values of the non-coded levels are included between 115.9 < P < 284.1 bars, 33.2 < T < 66.8 °C, and 6.4 < M < 73.6 wt%.

**Table 8:** Characterization of polar molecules fraction by amount extracted from SCG and content in the extract of Caffeine, 3-CaffeoylQuinic Acid (3-CQA) and Total Hydroxycinnamic Acids (THA) obtained with HPLC-DAD for our study experiments and in the literature.

Experiments	Hydroalcoholic Reference (EtOH + H_2_O)	Pure Water (H_2_O)	RYP Optimization (SC-CO_2_ + H_2_O)	RYL + RYP Optimization (SC-CO_2_ + H_2_O)	No Water (SC-CO_2_)
Caffeine recovery yield (wt%)	100	94.82	65.97	65.66	0.75
Caffeine content (g_CAF_·100 g^−1^_extract_)	8.33	8.83	100.05	99.93	30.63
Caffeine concentration (mg_CAF_·g^−1^_SCG_)	8.66	8.22	5.72	5.69	0.06
3-CQA content (g_CQA_·100 g^−1^_extract_)	2.69	2.57	n.d	n.d	n.d
3-CQA concentration (mg_CQA_·g^−1^_SCG_)	2.80	2.67	n.d	n.d	n.d
Total Hydroxycinnamic Acids (THA) content (g_CQA_·100 g^−1^_extract_)	9.54	8.48	0.59	0.79	0.34
Total Hydroxycinnamic Acids (THA) concentration (mg_CQA_·g^−1^_SCG_)	9.92	8.82	0.06	0.08	0.04

n.d. = Not detected.

## Data Availability

The datasets generated for this study are available on request to the corresponding author.

## Supplementary Materials

The following supporting information can be downloaded at: https://www.mdpi.com/article/10.3390/foods11244089/s1, Figure S1: Supercritical CO_2_ apparatus scheme from Top Industries (serial number 3133 0000) equipped with a carbon dioxide cylinder with plunging tube (≥99.7 %CO_2_, 34 kgs, Air Liquide, France), a dosing pump with Coriolis debitmeter (0–150 g_CO2_·min^−1^, HP Flow 50–1000, serial number 2776 5000), a cooling system set between 0–3 °C (Proficool Genius, Germany), a pre-heater with electric heating resistors, a autoclave extractor (500 mL, 600 bars, 150 °C), homemade cellule of extraction (Aluminium, sintered metal disk, Teflon seal), an Automatic Back Pressure Regulator ABPR (689.48 bars, Premier 3000AL, Premier Industries, USA) set with compressed air at 100 psi or 6.89 bars, an autoclave separator (250 mL, 200 bars, 150 °C), bursting disks (650 bars, Sitec, Switzerland) and a touchpad to control the supercritical apparatus Monitouch TS1070Si. Figure S2: Normal probability plot of Recovery Yield of Lipids (RYL) for SC-CO_2_ extraction. Figure S3: Normal probability plot of Recovery Yield of Polyphenols (RYP) for SC-CO_2_ extraction. Figure S4: Spent coffee grounds obtained by supercritical CO_2_ extraction (left) and by n-hexane Soxhlet (right). Figure S5. Chromatogram at λ = 325 nm of hydroalcoholic extract obtained from spent coffee grounds. Experimental conditions of extraction: 2 g of defatted SCG with 50 mL of hydroalcoholic solution EtOH/H_2_O (40/60 % *v*/*v*) at 60 °C for 15 min (reference method for polar molecules extraction). Figure S6. UV-Visible spectra of the peaks from the chromatogram at λ = 325 nm of hydroalcoholic extract obtained from spent coffee grounds. Experimental conditions of extraction: 2 g of defatted SCG with 50 mL of hydroalcoholic solution EtOH/H_2_O (40/60 % *v*/*v*) at 60 °C for 15 min (reference method for polar molecules extraction). Figure S7. Chromatogram at λ = 273 nm of hydroalcoholic extract obtained from spent coffee grounds. Experimental conditions of extraction: 2 g of defatted SCG with 50 mL of hydroalcoholic solution EtOH/H_2_O (40/60 % *v/v*) at 60 °C for 15 min (reference method for polar molecules extraction). Figure S8. Chromatogram at λ = 325 nm of supercritical CO_2_ extract (hydroalcoholic fraction) obtained from spent coffee grounds. Experimental conditions of extraction: SC-CO_2_ with P = 265 bars, T = 55 °C and moisture content 55wt% (optimized SC-CO_2_ method for apolar and polar molecules). Figure S9. Chromatogram at λ = 273 nm of supercritical CO_2_ extract (hydroalcoholic fraction) obtained from spent coffee grounds. Experimental conditions of extraction: SC-CO_2_ with P = 265 bars, T = 55 °C and moisture content 55wt% (optimized SC-CO_2_ method for apolar and polar molecules. Table S1. Experimental and predicted data on Recovery Yield of Lipids and Polyphenols of SC-CO_2_ extraction of SCG under different conditions of pressure (bars), temperature (°C) and moisture content (wt%) based on central composite rotatable design (CCRD) for response surface analysis. Experimental data of Absolute Yield of Lipids and Polyphenols of reference method (A, B) and experiments are available in this table. Reference [98] are cited in the Supplementary Materials.

## Abbreviations

[C_4_C_1_im][PF_6_]	1-Butyl-3-methylimidazolium hexafluorophosphate
3-CQA	3-CaffeoylQuinic Acid
Adj SS	Adjusted Sum Square
ANOVA	ANalysis OF VAriance
AOAC	Association of Official Agricultural Chemists
AOC	AntiOxidant Capacity
AV	Acid Value
CCD	Central Composite Design
CCRD	Central Composite Rotatable Design
DES	Deep Eutectic Solvent
DF	Desirability Function
DoE	Design of Experiments
DPPH	2,2-Diphenyl-1-PicrylHydrazyl
DY	Drying Yield
EV	Ester Value
EY	Extraction Yield
FAL	Fatty Acids Level
FAME	Fatty Acids Methyl Ester
FAP	Fatty Acids Profile
FFA	Free Fatty Acids
F-value	Fisher value
GC-MS	Gas Chromatography—Mass Spectrometry
HPLC-DAD	High Performance Liquid Chromatography—Diode Array Detector
HVED	High Voltage Electric Discharge
IL	Ionic Liquid
ISO	International Organization for Standardization
IV	Iodine Value
M	Moisture content
MW	MicroWave
NADES	Natural Deep Eutectic Solvent
P	Pressure
PEF	Pulse Electric Field
PES	PolyEtherSulfone
RESS	Rapid Expansion of Supercritical Solution
RSM	Response Surface Methodology
RYL	Recovery Yield of Lipids
RYP	Recovery Yield of Polar molecules
SC-CO_2_	Supercritical Carbon Dioxide
SCG	Spent Coffee Grounds
SCW	SubCritical Water
SV	Saponification Value
T	Temperature
TAG	TriAcylGlycerol
THA	Total Hydroxycinnamic Acids
US	Ultrasound
