# Supplementary material for: Supercritical Carbon Dioxide in Presence of Water for the Valorization of Spent Coffee Grounds: Optimization by Response Surface Methodology and Investigation of Caffeine Extraction Mechanism
Source: Foods. 2022 Dec 17;11(24):4089. doi: 10.3390/foods11244089 (PMC9777831; doi:10.3390/foods11244089)
Supplement: Supplementary file 1 [file foods-11-04089-s001.zip › foods-2092694-supplementary.pdf]

Supplementary File:

GC-MS Analyses of qualitative and quantitative fatty acids present in lipids fraction

GC-MS analyses were performed with an Agilent (PaloAlto, CA, USA) 6890 Series gas chromatograph mass spectrometer equipped with a 7673 Series injector and a 5973 Network Mass Selective Detector. One microliter of the FAME solution with C15:0 internal standard was injected in splitless mode. The inlet temperature was 250 °C and carrier gas was pure helium (≥ 99.999 %). Separation were achieved a 30 m Optima 5 MS 95% DiMethylPolySiloxane capillary column (Macherey-Nagel, FRANCE) cross-linked with 5% of DiPhenyl (0.250 mm i.d., 0.30 m film thickness). The GC oven temperature was kept at 50 °C for the first minute, then increased to 300 °C at 10 °C.min^{-1} and kept at 300 °C for 15 min. The flow rate of helium flow was 1 mL.min-1. Detection was performed in scan mode between m/z: 35 and 500. MS spectra were obtained at interface temperature 250°C, ion source temperature 150°C and ionization energy of 70 eV. The software used for the control of the GC-MS and data acquisition was Enhanced ChemStation G1701CA Version C.00.00.21-Dec-1999. Based on this analysis, Fatty Acids Profile (FAP) and Fatty Acids Level (FAL) were calculated (Equation S1, Equation S2):

Equation S1. Fatty Acids Profile (FAP)

$$\boldsymbol{Fatty\ Acids\ Profile\ (FAP)} = \frac{mass\ of\ Cx{:}y\ (mol)}{mass\ of\ all\ fatty\ acids\ (mol)} \times 100$$

Equation S2. Fatty Acids Level (FAL)

$$\boldsymbol{Fatty\ Acids\ Level\ (FAL)} = \frac{mass\ of\ all\ fatty\ acids\ (g)}{mass\ of\ oil\ (g)} \times 100$$

Acid, Saponification, Ester and Iodine Values (AV, SV, EV, IV)

Acid Value (AV) was measured using the reference method from the ISO 660 protocol [1,2]. AV was calculated by using (Eq. S3):

Equation S3. Acid Value (AV)

$$\boldsymbol{Acid\ Value\ (AV)} = \frac{M_{KOH}\ (mol.L^{-1}) \times V_{KOH}\ (mL) \times N_{KOH}\ (mol.L^{-1})}{W_{oil}\ (g)}\ in\ mg_{KOH}\cdot g_{oil}^{-1}$$

Saponification Value (SV) was measured with reference method from the A.O.A.C 920.160 protocol [2,3]. SV was calculated by using (Eq. S4):

Equation S4. Saponification Value (SV)

$$\textbf{Saponification Value (SV)} = \frac{M_{KOH}\ (mol.L^{-1}) \times (V_{HCl\ Blank} - V_{HCl\ Sample})\ (mL) \times N_{HCl}\ (mol.L^{-1})}{W_{oil}\ (g)}\ in\ mg_{KOH}.g_{oil}^{-1}$$

Ester Value (EV) and wt% Free Fatty Acids (%FFA) were calculated according to the following equations (Eq. S5, Eq. S6).

Equation S5. Ester Value (EV)

$$\textbf{Ester Value (EV)} = SV - AV\ in\ mg_{KOH}.g_{oil}^{-1}$$

Equation S6. Wt% Free Fatty Acids (%FFA)

$$wt\%\ \textbf{Free Fatty Acids}\ (\%\textbf{FFA}) = \frac{AV}{SV}\ in\ wt\%$$

Iodine value (IV) was measured with the reference method according to A.O.A.C 920.159 protocol, modified by using cyclohexane instead of carbon tetrachloride [2,4,5]. IV was calculated by using (Eq. S7):

Equation S7. Iodine value (IV)

$$\textbf{Iodine Value (IV)} = \frac{\frac{M_I}{10}(mol.100mL^{-1}) \times (V_{Na_2S_2O_3\ Blank} - V_{Na_2S_2O_3\ Sample})\ (mL) \times N_{Na_2S_2O}}{W_{oil}\ (g)}$$

HPLC-DAD Analyses of caffeine and phenolic compounds present in polar molecules fraction

The quantification of caffeine, 3-caffeoylquinic acid (3-CQA) and Total Hydroxycinnamic Acids (THA) was performed using a Waters 600 HPLC-DAD system (Waters 600) equipped with controller (Waters 600), autosampler (Waters 717Plus), degasser (Waters), quaternary pump (Waters 600) and Photodiode Array Detector (Waters 996). Separation was achieved using an NUCLEOSIL C18 PAH column (150 mm× 4.6 mm, 2.6 µm, Macherey Nagel, France) maintained at 40 °C (Oven Controller 480 Kontron Instrument). The mobile phase was composed of eluent A (20 mM KH2PO4 buffer containing 0.1% phosphoric acid) and eluent B (acetonitrile containing 0.1% phosphoric acid); the gradient program was: 0–5 min, 3% B (v/v); 5–15 min, 7% B (v/v); 15–25 min, 7% B (v/v); 25–45 min, 25% B (v/v); 45–50 min, 3% B (v/v); and 50–55 min, 3% B (v/v). The flow rate and injection volume were 1 mL.min-1 and 10 µL, respectively. The identification of hydroxycinnamic acids was performed with wavelength scan between λ = 200 and 400 nm. The quantification of the THA was expressed in equivalent of 3-CQA, a bonded monohydroxycinnamic acid as caffeic acid with similar maximal UV-Visible absorption than non-bonded caffeic acid was performed at λ = 320-323 nm [6]. The quantification of 3-CQA and THA was realized at a wavelength at λ = 325 nm. The detection and quantification of caffeine was realized at a wavelength at λ = 273 nm. The software used for the control of the HPLC-DAD and data acquisition was Empower 2 Software.

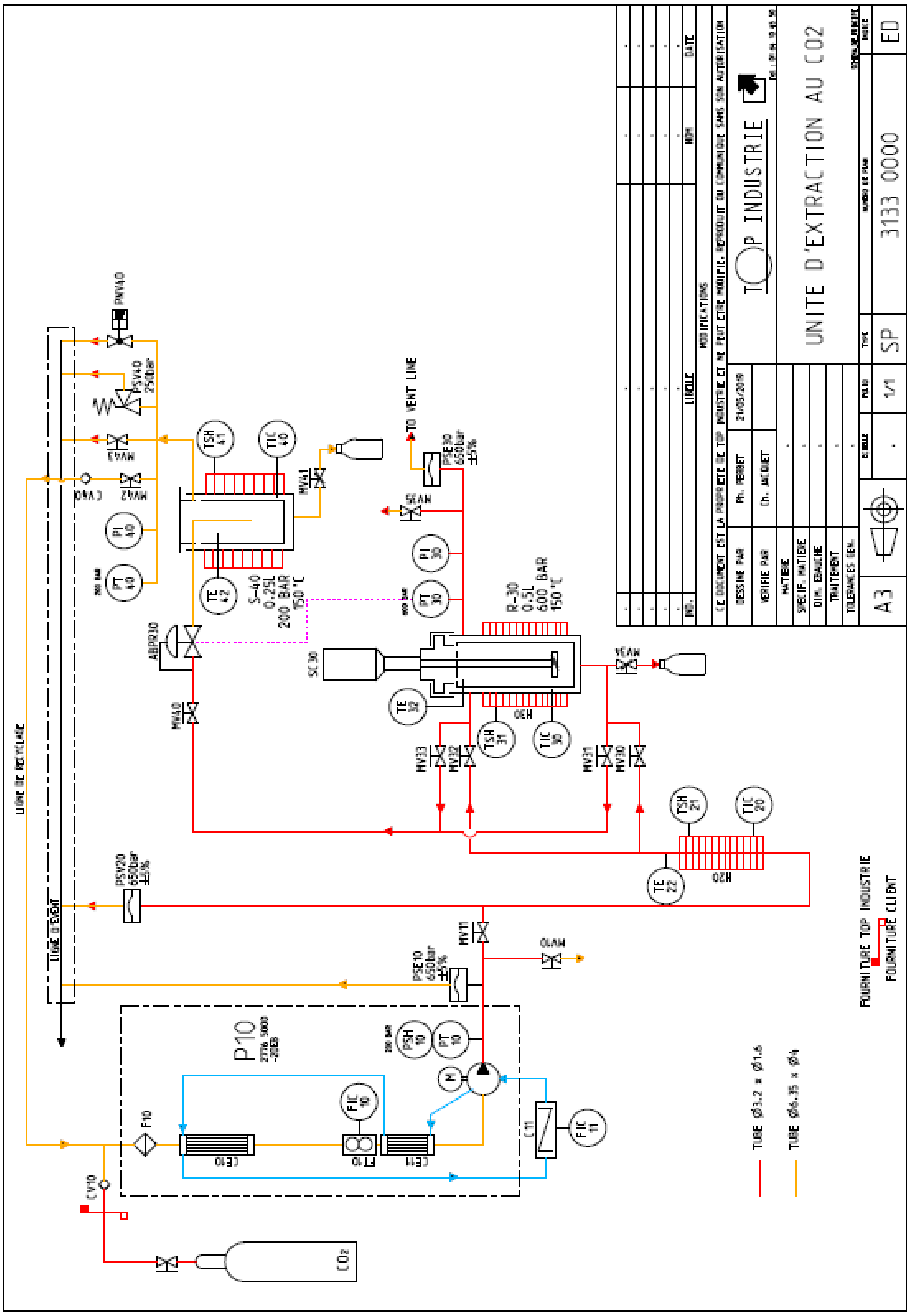

Figure S1. Supercritical CO_2 apparatus scheme from Top Industries (serial number 3133 0000) equipped with a carbon dioxide cylinder with plunging tube (≥99.7 %CO2, 34 kgs, Air Liquide, France), a dosing pump with Coriolis debitmeter (0-150 $g_{CO2}.min^{-1}$, HP Flow 50-1000, serial number 2776 5000), a cooling system set between 0-3 °C (Proficool Genius, Germany), a pre-heater with electric heating resistors, a autoclave extractor (500 mL, 600 bars, 150 °C), homemade cellule of extraction (Aluminium, sintered metal disk, Teflon seal), an Automatic Back Pressure Regulator ABPR (689.48 bars, Premier 3000AL, Premier Industries, USA) set with compressed air at 100 psi or 6.89 bars, an autoclave separator (250 mL, 200 bars, 150 °C), bursting disks (650 bars, Sitec, Switzerland) and a touchpad to control the supercritical apparatus Monitouch TS1070Si.

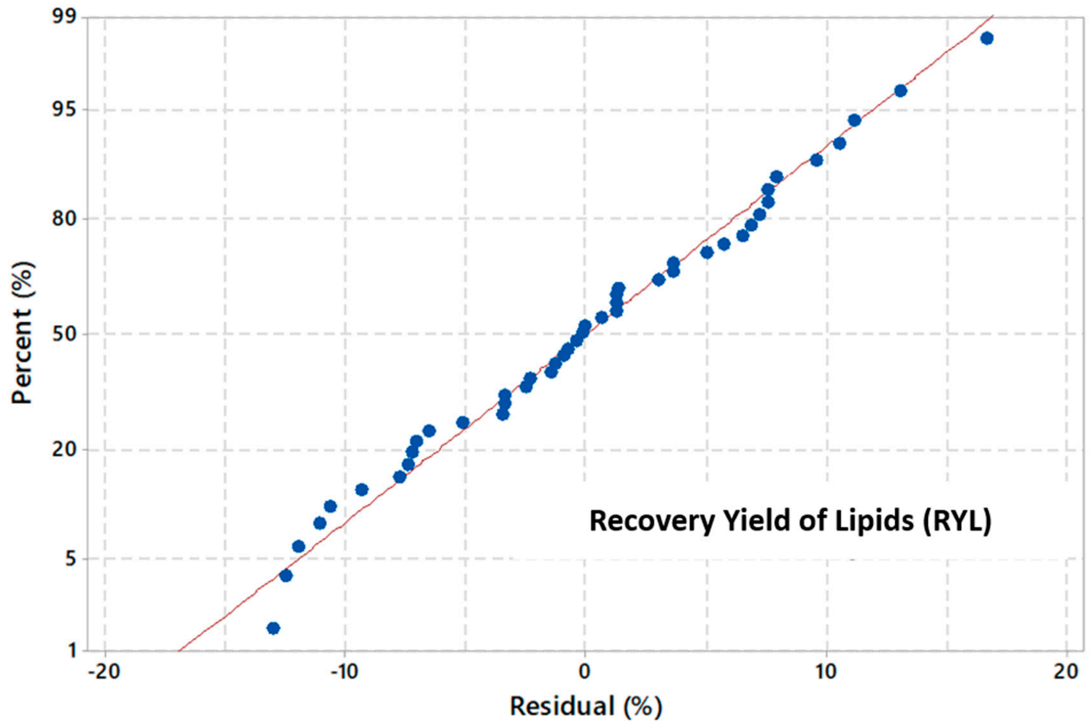

Figure S2. Normal probability plot of Recovery Yield of Lipids (RYL) for SC-CO_2 extraction

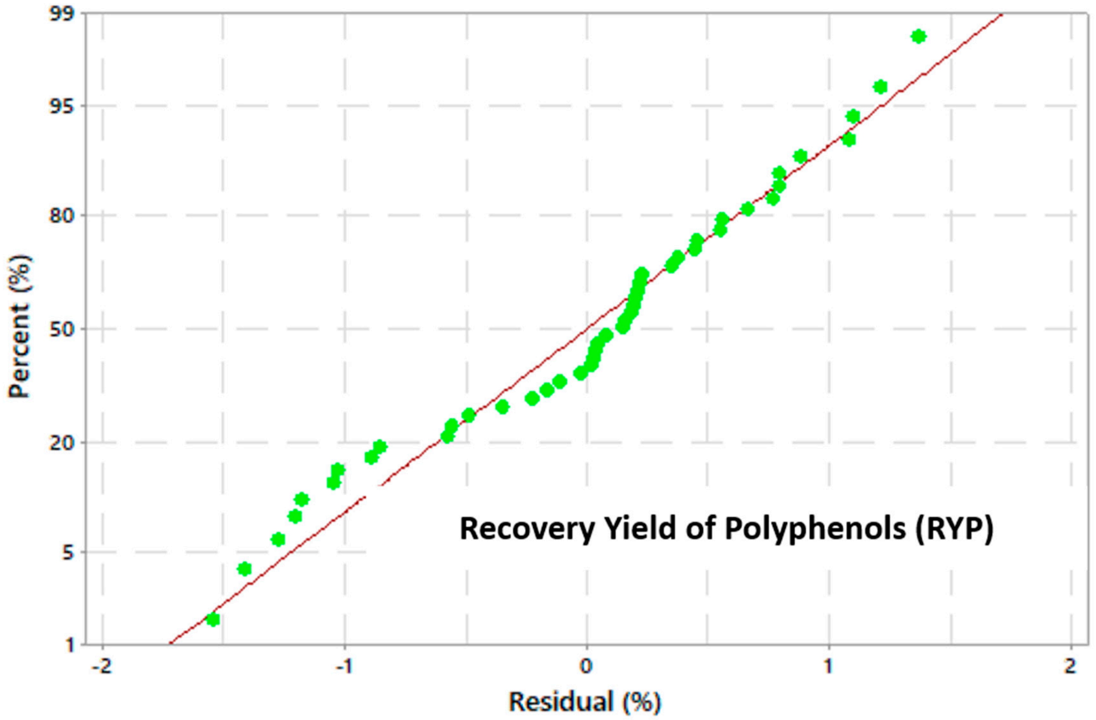

Figure S3. Normal probability plot of Recovery Yield of Polyphenols (RYP) for SC-CO_2 extraction.

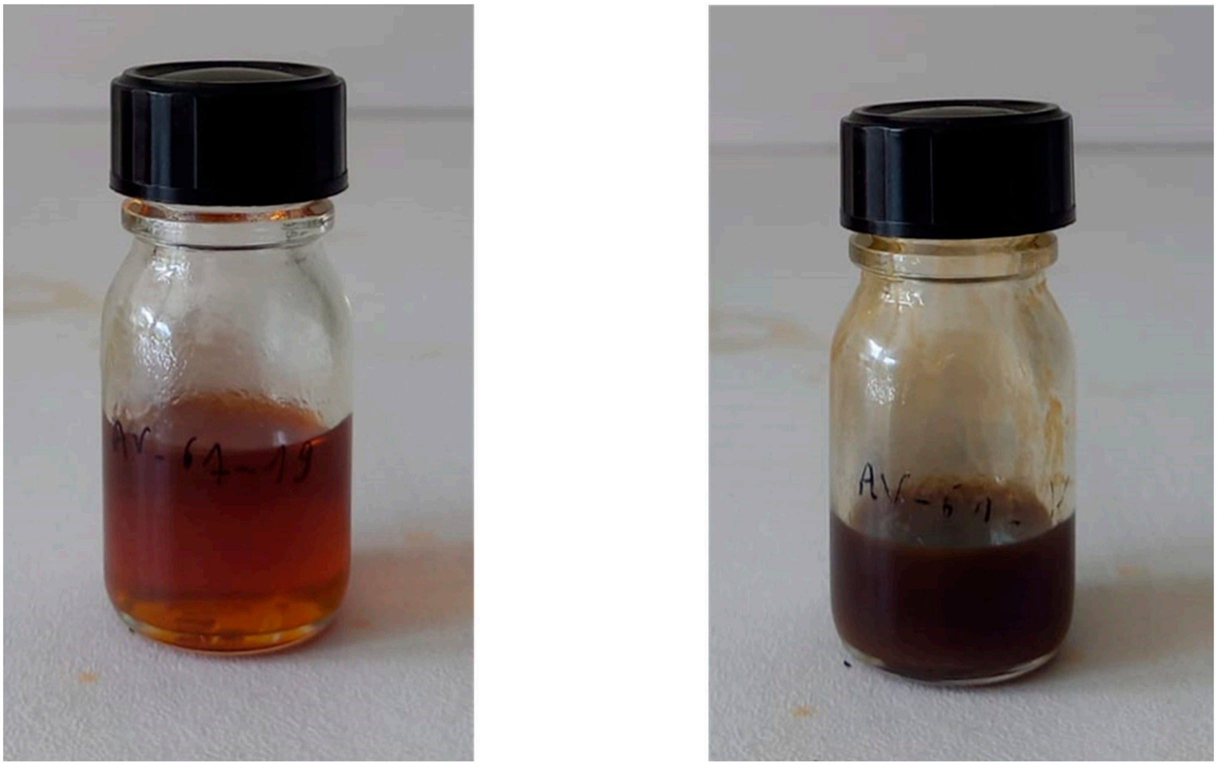

Figure S4. Spent coffee grounds obtained by supercritical CO_2 extraction (left) and by n-hexane Soxhlet (right)

Table S1. Experimental and predicted data on Recovery Yield of Lipids and Polyphenols of SC-CO_2 extraction of SCG under different conditions of pressure (bars), temperature (°C) and moisture content (wt%) based on central composite rotatable design (CCRD) for response surface analysis. Experimental data of Absolute Yield of Lipids and Polyphenols of reference method (A, B) and experiments are available in this table.

	**Independent variables**			**Response variables**					
**Experience**	**Pressure (bars)**	**Temperature (°C)**	**Moisture content (wt%)**	**Recovery Yield of Lipids (wt%)**		**Recovery Yield of Polyphenols (wt%)**		**Absolute Yield of Lipids ($g_{lipids}.100g^{-1}_{drySCG}$)**	**Absolute Yield of Polyphenols ($g_{polyphenols}.100g^{-1}_{drySCG}$)**
				Measured	Predicted	Measured	Predicted	Measured	Measured
**1**	150.00 (-1)[a]	60.00 (+1)	60.00 (+1)	31.27	31.69	4.94	5.11	3.84	0.5139
**2**	115.91 (-α)	50.00 (0)	40.00 (0)	**9.76**	19.11	3.74	4.95	**1.20**	0.3887
**3**	200.00 (0)	33.18 (-α)	40.00 (0)	86.84	99.29	5.06	6.10	10.68	0.5268
**4**	200.00 (0)	50.00 (0)	73.64 (+α)	76.82	79.18	5.32	4.98	9.44	0.5538
**5**	250.00 (+1)	60.00 (+1)	60.00 (+1)	86.21	96.86	5.65	6.00	10.60	0.5878
**6**	150.00 (-1)	40.00 (-1)	20.00 (-1)	85.33	77.49	4.88	3.80	10.49	0.5077
**7**	200.00 (0)	50.00 (0)	40.00 (0)	90.36	90.47	6.15	6.00	11.11	0.6393
**8**	200.00 (0)	66.82 (+α)	40.00 (0)	83.80	74.30	6.48	6.28	10.30	0.6738
**9**	250.00 (+1)	60.00 (+1)	20.00 (-1)	91.56	99.30	4.27	4.25	11.26	0.4441
**10**	150.00 (-1)	60.00 (+1)	20.00 (-1)	29.40	32.83	5.08	4.71	3.62	0.5285
**11**	200.00 (0)	66.82 (+α)	40.00 (0)	90.83	74.30	6.46	6.28	11.17	0.6721
**12**	284.09 (+α)	50.00 (0)	40.00 (0)	91.19	83.67	5.89	5.68	11.21	0.6131
**13**	284.09 (+α)	50.00 (0)	40.00 (0)	90.85	83.67	5.56	5.68	11.17	0.5781
**14**	150.00 (-1)	40.00 (-1)	60.00 (+1)	82.72	71.62	6.47	5.37	10.17	0.6731
**15**	115.91 (-α)	50.00 (0)	40.00 (0)	12.04	19.11	4.39	4.95	1.48	0.4562
**16**	250.00 (+1)	60.00 (+1)	60.00 (+1)	89.61	96.86	5.77	6.00	11.02	0.6004
**17**	250.00 (+1)	60.00 (+1)	60.00 (+1)	90.32	96.86	5.14	6.00	11.11	0.5350
**18**	250.00 (+1)	40.00 (-1)	20.00 (-1)	90.28	89.09	4.55	3.78	11.10	0.4728
**19**	150.00 (-1)	60.00 (+1)	60.00 (+1)	30.75	31.69	4.62	5.11	3.78	0.4805
**20**	200.00 (0)	33.18 (-α)	40.00 (0)	86.33	99.29	4.92	6.10	10.61	0.5119
**21**	150.00 (-1)	60.00 (+1)	20.00 (-1)	27.71	32.83	5.92	6.28	3.41	0.6159
**22**	150.00 (-1)	60.00 (+1)	20.00 (-1)	30.29	32.83	5.50	6.28	3.72	0.5722
**23**	200.00 (0)	50.00 (0)	73.64 (+α)	77.84	79.18	5.52	4.98	9.57	0.5744
**24**	200.00 (0)	50.00 (0)	40.00 (0)	89.70	90.47	6.22	6.00	11.03	0.6469
**25**	150.00 (-1)	40.00 (-1)	60.00 (+1)	78.07	71.62	5.82	5.37	9.60	0.6052
**26**	250.00 (+1)	40.00 (-1)	60.00 (+1)	84.91	81.92	6.67	6.70	10.44	0.6942
**27**	250.00 (+1)	60.00 (+1)	20.00 (-1)	88.20	99.30	4.28	4.25	10.84	0.4449
**28**	150.00 (-1)	40.00 (-1)	20.00 (-1)	87.93	77.49	5.17	3.80	10.81	0.5373
**29**	250.00 (+1)	40.00 (-1)	60.00 (+1)	83.11	81.92	**6.74**	6.70	10.22	**0.7009**
**30**	284.09 (+α)	50.00 (0)	40.00 (0)	90.49	83.67	6.23	5.68	11.13	0.6481
**31**	150.00 (-1)	40.00 (-1)	60.00 (+1)	79.11	71.62	5.56	5.37	9.73	0.5779
**32**	150.00 (-1)	60.00 (+1)	60.00 (+1)	28.17	31.69	5.26	5.11	3.46	0.5472
**33**	115.91 (-α)	50.00 (0)	40.00 (0)	15.73	19.11	3.53	4.95	1.93	0.3670
**34**	200.00 (0)	50.00 (0)	40.00 (0)	91.06	90.47	6.07	6.00	11.20	0.6318
**35**	200.00 (0)	50.00 (0)	6.36 (-α)	89.73	86.17	1.13	2.18	11.03	0.1180
**36**	150.00 (-1)	40.00 (-1)	20.00 (-1)	82.42	77.49	4.60	3.80	10.13	0.4780
**37**	250.00 (+1)	40.00 (-1)	60.00 (+1)	87.61	81.92	6.12	6.70	10.77	0.6366
**38**	200.00 (0)[a]	50.00 (0)	6.36 (-α)	84.67	86.17	**0.64**	2.18	10.41	**0.0665**
**39**	200.00 (0)	50.00 (0)	6.36 (-α)	89.74	86.17	0.91	2.18	11.03	0.0945
**40**	200.00 (0)	66.82 (+α)	40.00 (0)	87.32	74.30	6.47	6.28	10.74	0.6729
**41**	250.00 (+1)	40.00 (-1)	20.00 (-1)	90.36	89.09	4.66	3.78	11.11	0.4845
**42**	250.00 (+1)	40.00 (-1)	20.00 (-1)	90.32	89.09	4.43	3.78	11.10	0.4611
**43**	250.00 (+1)	60.00 (+1)	20.00 (-1)	**91.89**	99.30	4.29	4.25	**11.30**	0.4457
**44**	200.00 (0)	50.00 (0)	73.64 (+α)	78.98	79.18	5.42	4.98	9.71	0.5641
**45**	200.00 (0)	33.18 (-α)	40.00 (0)	87.36	99.29	5.21	6.10	10.74	0.5417
				**Reference methods**					
**A**	Soxhlet *n*-hexane, 10 g, 4 hours			100	/	/	/	12.29	/
**B**	Hydroalcoholic solution EtOH/H_2O (40/60 v/v), 10g, 50 mL, 60 °C, 15 min, 3 times			/	/	100	/	/	10.40

[a] Number in parentheses represent the level of independent parameters; [b] Min and max values are given in bold.

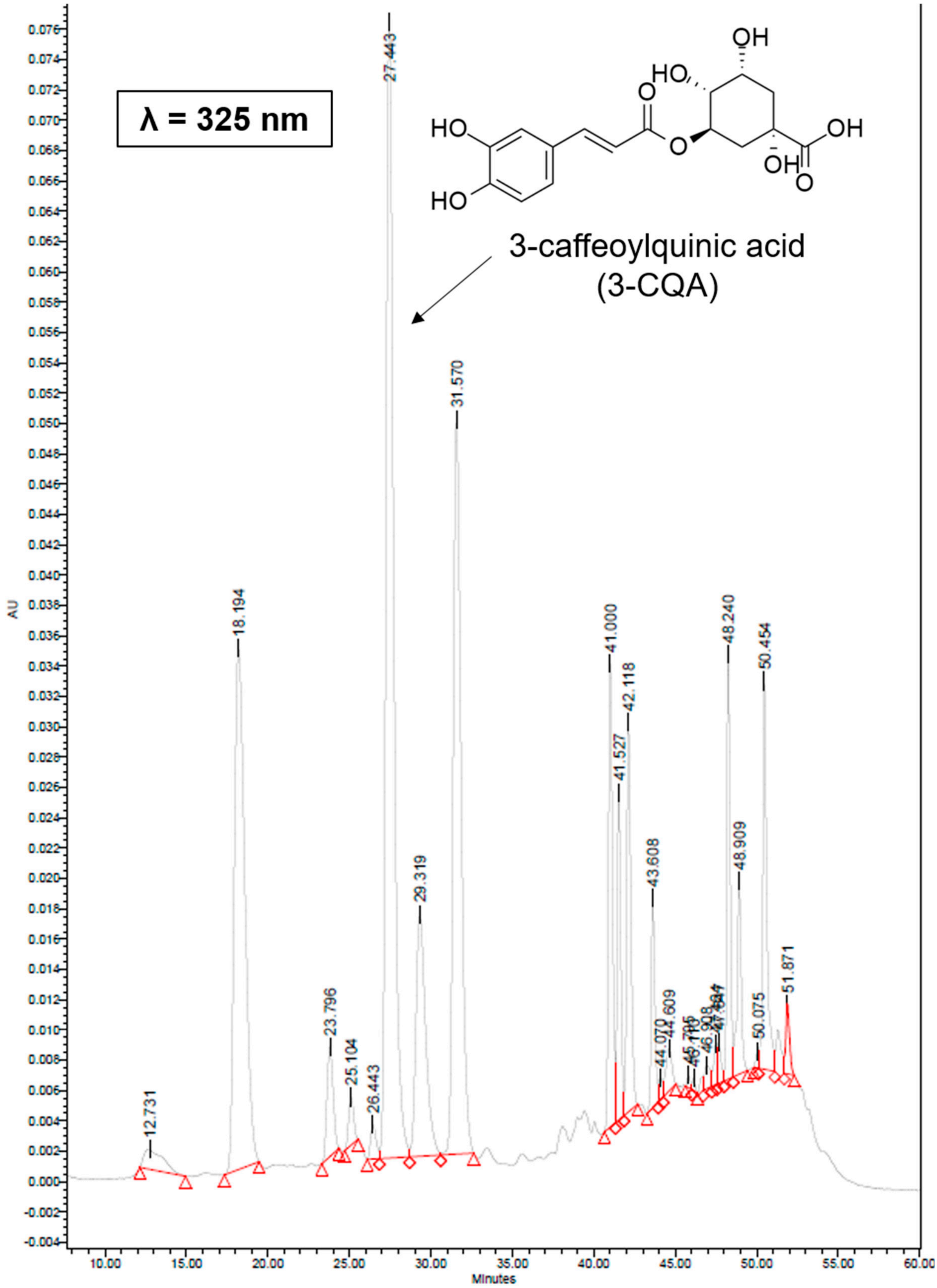

Figure S5. Chromatogram at λ = 325 nm of hydroalcoholic extract obtained from spent coffee grounds. Experimental conditions of extraction: 2g of defatted SCG with 50 mL of hydroalcoholic solution $EtOH/H_2O$ (40/60%, *v*/*v*) at 60 °C for 15 min (reference method for polar molecules extraction).

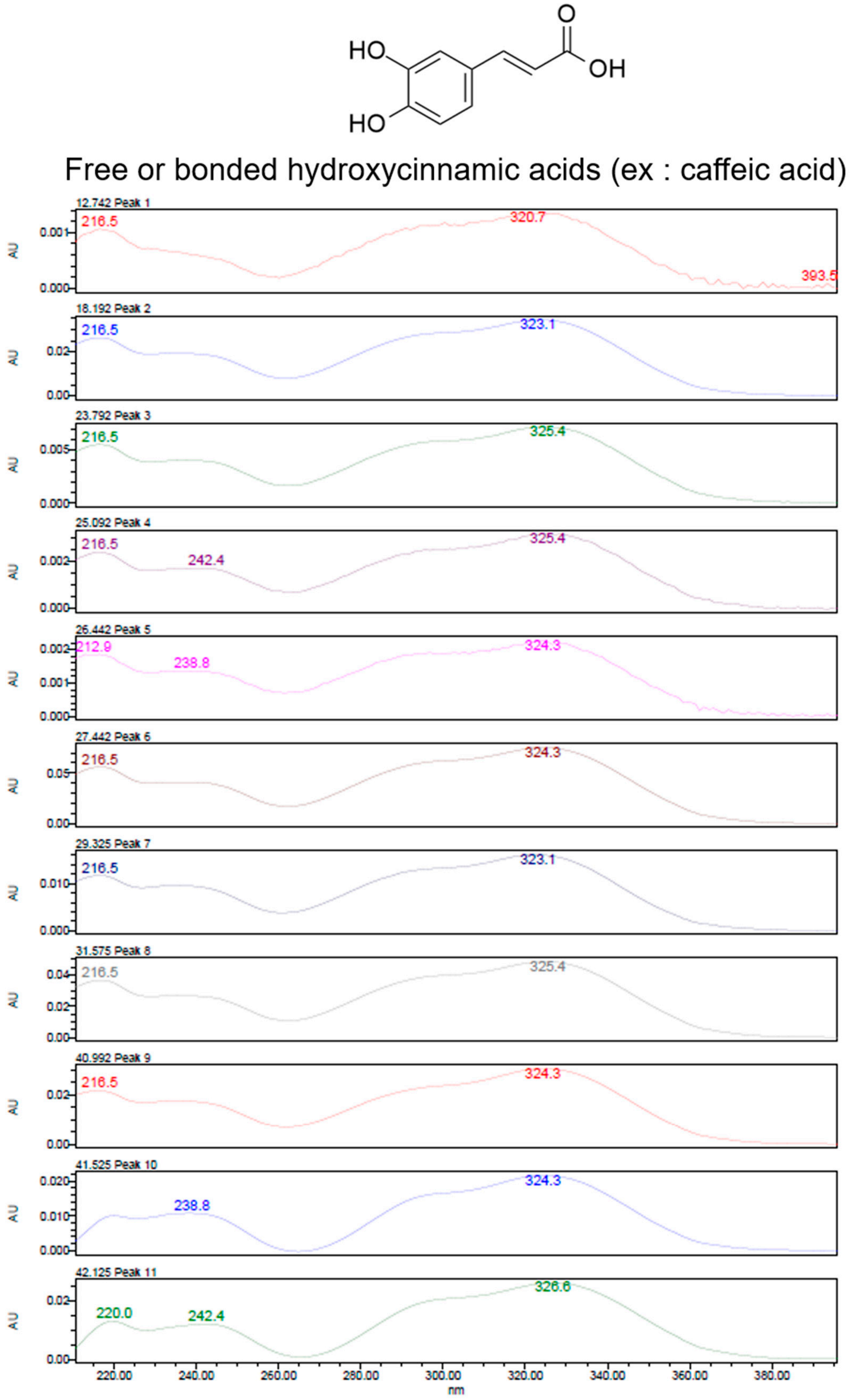

Figure S6. UV-Visible spectra of the peaks from the chromatogram at **λ** = 325 nm of hydroalcoholic extract obtained from spent coffee grounds. Experimental conditions of extraction: 2g of defatted SCG with 50 mL of hydroalcoholic solution EtOH/H_2O (40/60 % v/v) at 60 °C for 15 min (reference method for polar molecules extraction).

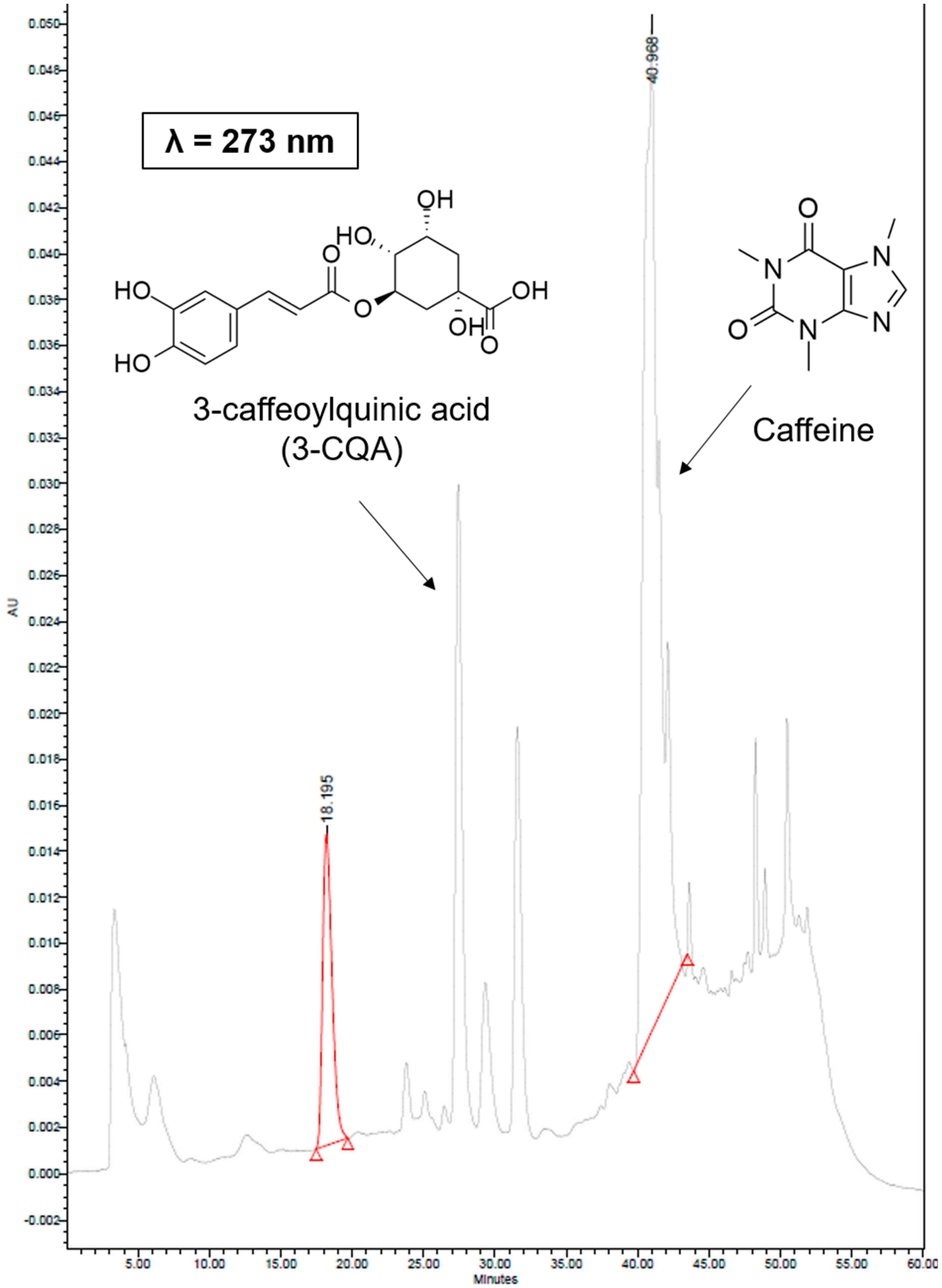

Figure S7. Chromatogram at λ = 273 nm of hydroalcoholic extract obtained from spent coffee grounds. Experimental conditions of extraction: 2g of defatted SCG with 50 mL of hydroalcoholic solution EtOH/H_2O (40/60 % v/v) at 60 °C for 15 min (reference method for polar molecules extraction).

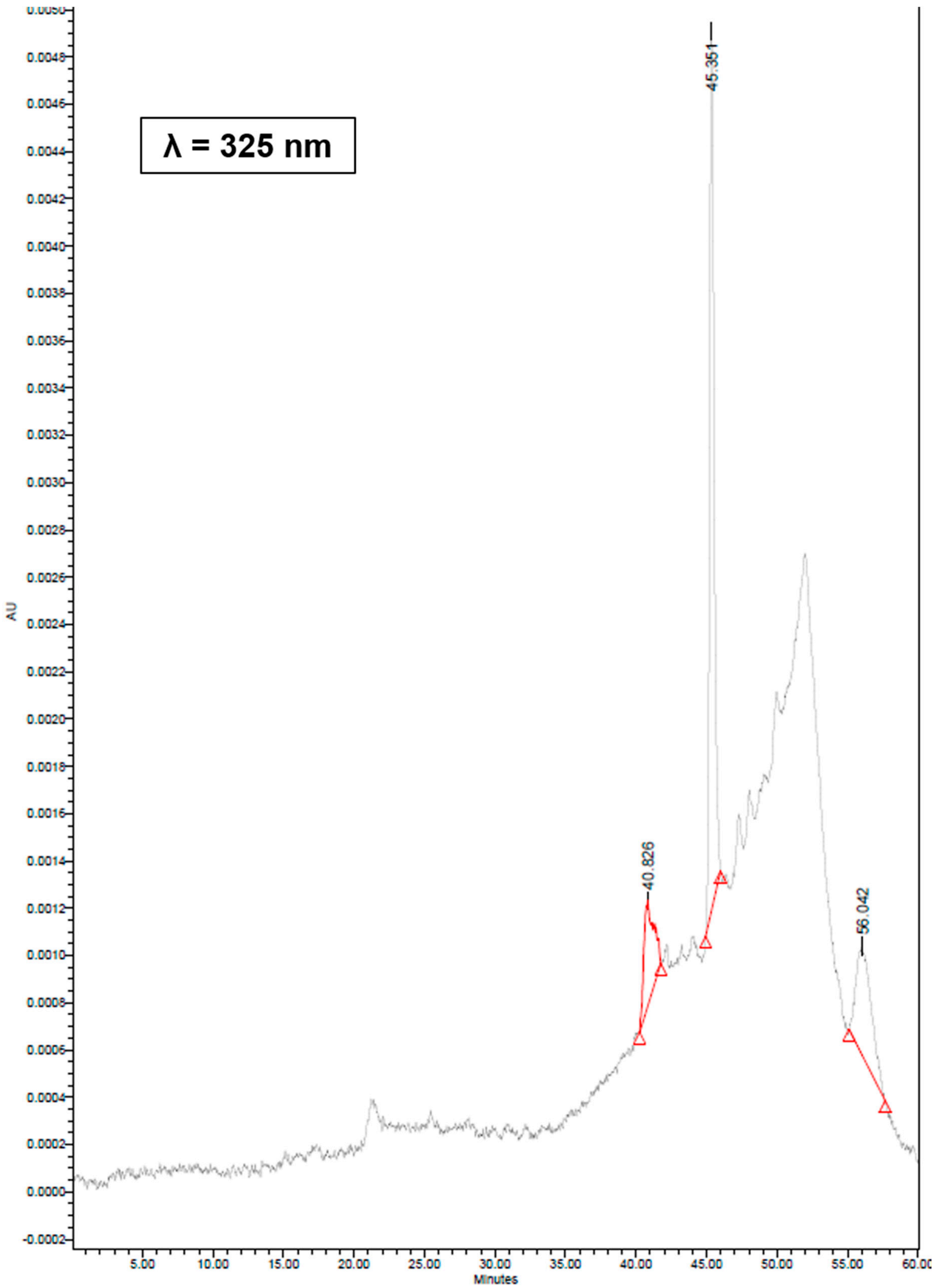

Figure S8. Chromatogram at λ = 325 nm of supercritical CO_2 extract (hydroalcoholic fraction) obtained from spent coffee grounds. Experimental conditions of extraction: SC-CO_2 with P = 265 bars, T = 55 °C and moisture content 55wt% (optimized SC-CO_2 method for apolar and polar molecules).

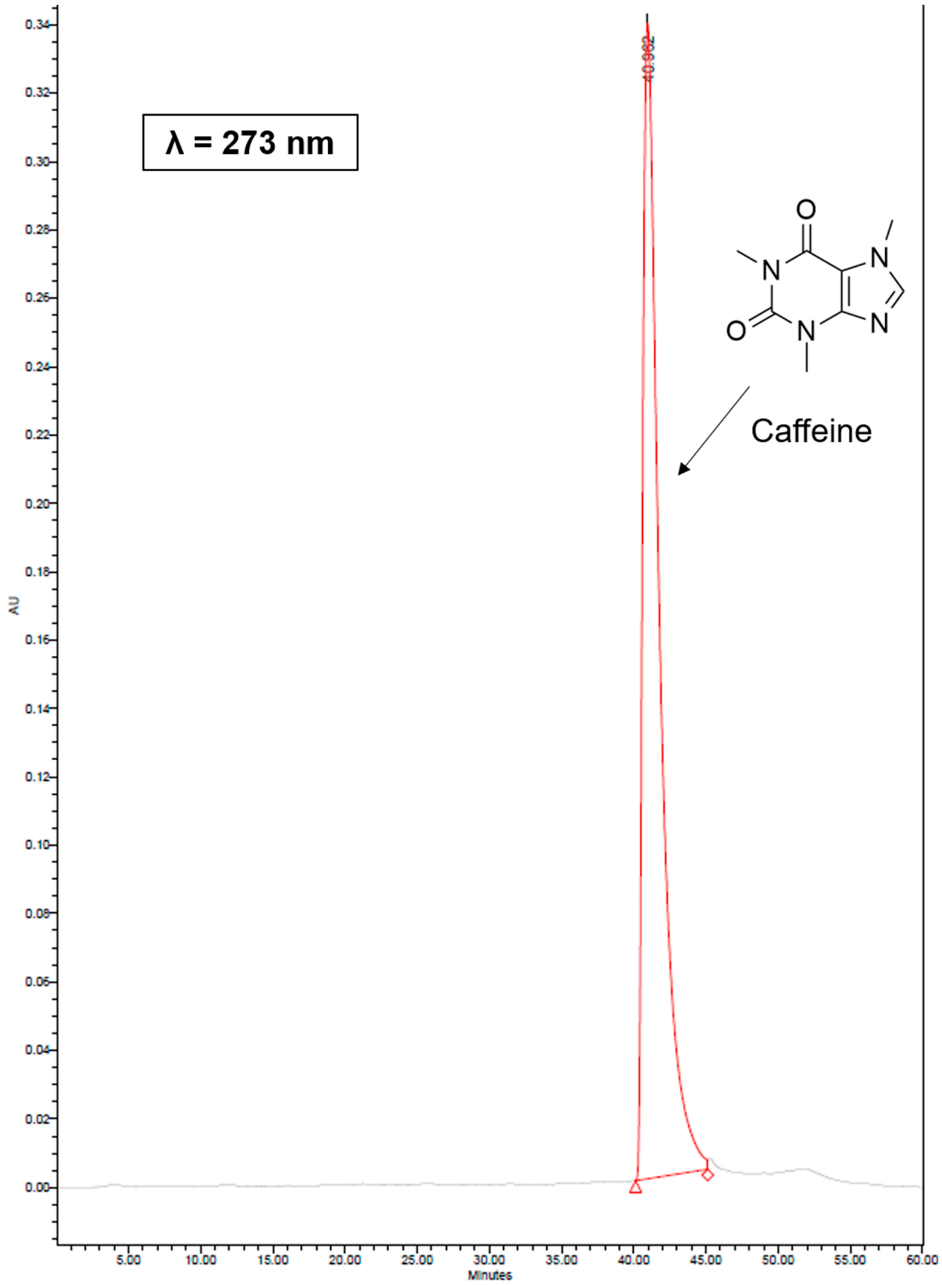

Figure S9. Chromatogram at **λ** = 273 nm of supercritical CO_2 extract (hydroalcoholic fraction) obtained from spent coffee grounds. Experimental conditions of extraction: SC-CO_2 with P = 265 bars, T = 55 °C and moisture content 55wt% (optimized SC-CO_2 method for apolar and polar molecules.

References:

[1] ISO, Standard Method ISO 660: 1996, Animal and vegetable fats and oils-Determination of acid value and acidity, (1996).

[2] F.S.A.S.A. of I.M. of H. and F.W.G. of I. OILSANDFATS, Manual of Methods of Analysis of Foods Oils And Fats, (2012).

[3] A.O.A.C 920.160, AOAC Offic:al Method 920.160 Saponification Number, (1920).

[4] A.O.A.C 920.159, AOAC Official Method 920.159 - Iodine absorption number of oils and fats, (1920).

[5] D. Firestone, Determination of the iodine value of oils and fats: summary of collaborative study, Journal of AOAC International. 77 (1994) 674–676.

[6] M.N. Clifford, J. Wight, The measurement of feruloylquinic acids and caffeoylquinic acids in coffee beans. Development of the technique and its preliminary application to green coffee beans, Journal of the Science of Food and Agriculture. 27 (1976) 73–84.
